# Improved Detection of Potentially Pleiotropic Genes in Coronary Artery Disease and Chronic Kidney Disease Using GWAS Summary Statistics

**DOI:** 10.3389/fgene.2020.592461

**Published:** 2020-12-03

**Authors:** Haimiao Chen, Ting Wang, Jinna Yang, Shuiping Huang, Ping Zeng

**Affiliations:** ^1^Department of Epidemiology and Biostatistics, School of Public Health, Xuzhou Medical University, Xuzhou, China; ^2^Department of Infectious Diseases, People’s Hospital of Zhuji, Shaoxing, China; ^3^Center for Medical Statistics and Data Analysis, School of Public Health, Xuzhou Medical University, Xuzhou, China

**Keywords:** coronary artery disease, chronic kidney disease, pleiotropy-informed integrative analysis, gene-based association analysis, pleiotropic gene, genome-wide association study

## Abstract

The coexistence of coronary artery disease (CAD) and chronic kidney disease (CKD) implies overlapped genetic foundation. However, the common genetic determination between the two diseases remains largely unknown. Relying on summary statistics publicly available from large scale genome-wide association studies (*n* = 184,305 for CAD and *n* = 567,460 for CKD), we observed significant positive genetic correlation between CAD and CKD (*r*_*g*_ = 0.173, *p* = 0.024) via the linkage disequilibrium score regression. Next, we implemented gene-based association analysis for each disease through MAGMA (Multi-marker Analysis of GenoMic Annotation) and detected 763 and 827 genes associated with CAD or CKD (FDR < 0.05). Among those 72 genes were shared between the two diseases. Furthermore, by integrating the overlapped genetic information between CAD and CKD, we implemented two pleiotropy-informed informatics approaches including cFDR (conditional false discovery rate) and GPA (Genetic analysis incorporating Pleiotropy and Annotation), and identified 169 and 504 shared genes (FDR < 0.05), of which 121 genes were simultaneously discovered by cFDR and GPA. Importantly, we found 11 potentially new pleiotropic genes related to both CAD and CKD (i.e., *ARHGEF19, RSG1, NDST2, CAMK2G, VCL, LRP10, RBM23, USP10, WNT9B, GOSR2*, and *RPRML*). Five of the newly identified pleiotropic genes were further repeated via an additional dataset CAD available from UK Biobank. Our functional enrichment analysis showed that those pleiotropic genes were enriched in diverse relevant pathway processes including quaternary ammonium group transmembrane transporter, dopamine transport. Overall, this study identifies common genetic architectures overlapped between CAD and CKD and will help to advance understanding of the molecular mechanisms underlying the comorbidity of the two diseases.

## Introduction

Both coronary artery disease (CAD) and chronic kidney disease (CKD) are the leading causes of death and disability worldwide, representing serious global public health threats (Kessler et al., 2013; Inrig et al., 2014; Ene-Iordache et al., 2016; Levin et al., 2017; Musunuru and Kathiresan, 2019). In practice, it is often observed that CKD patients encounter an increased risk of CAD and CAD is in turn a major cause of death for CKD patients (Tonelli et al., 2012). Pathologically, the endothelial dysfunction is closely related to cardiovascular diseases and plays an important role in all stages of atherosclerosis (Ross, 1999). On the other hand, the role of CAD in CKD is also widely studied; for example, the endothelial dysfunction in the development of CKD was also well documented (Moody et al., 2012). As originally proposed by Lindner et al. (1974), CKD patients with an estimated glomerular filtration rate (eGFR) <60 ml/min per 1.73 m^2^ have 2∼16 times higher risk of major adverse cardiovascular events (MACE) compared to those with an eGFR > 60 ml/min per 1.73 m^2^ (Go et al., 2004). Moreover, for CKD patients not yet requiring renal replacement therapy, the probability of developing MACE is much higher than reaching end-stage renal disease (ESRD) and requiring renal replacement therapy (Foley et al., 2005).

All those empirical observations suggest that there exist a common susceptible mechanism underlying these two complex diseases. As part of efforts to understand their genetic foundation, in the past few years many large scale genome-wide association studies (GWASs) have been implemented for CAD (Nikpay et al., 2015) and CKD (Wuttke et al., 2019). It is found that a lot of genes and single nucleotide polymorphisms (SNPs) exhibit pleiotropic effects and are associated with both the two diseases (Solovieff et al., 2013; Supplementary Table 1). This genetic overlap partly contributes to the co-existence of CAD and CKD. The understanding of common genetic determinants has significant implication for identifying important biomarkers and developing novel therapeutic strategies for joint prediction, prevention, and intervention of CAD and CKD.

However, like many other diseases/traits (Manolio et al., 2009; Eichler et al., 2010; Gusev et al., 2013; Girirajan, 2017; Kim et al., 2017; Young, 2019), CAD- or CKD-associated SNPs identified by GWAS only explain a very small fraction of phenotypic variance of CKD (Wuttke et al., 2019) and CAD (Nikpay et al., 2015), implying that a large number of genetic variants with small to modest effect sizes (but still important) have yet been discovered and that more pleiotropic genes would be found if increasing sample sizes (Wang et al., 2005; Altshuler et al., 2008; Tam et al., 2019). However, the increase of sample sizes is generally not feasible since the recruiting and genotyping of additional participants are time consuming and expensive. Therefore, it is a promising way to leverage genetic computational methods that can efficiently analyze information contained in the existing pool of available GWAS summary statistics for identifying loci with pleiotropic effects.

To achieve this aim, many pleiotropy-informed approaches have been proposed (Andreassen et al., 2013; Chung et al., 2014; Zeng et al., 2018). Those previous studies were focused on individual SNP associations and fine-mapping was further needed to find causal genes once newly novel genetic variants were detected (Hormozdiari et al., 2014, 2015; Wen et al., 2015; Kichaev et al., 2016). In addition, those methods cannot effectively handle the correlation among genetic variants due to linkage disequilibrium (LD) (Zeng et al., 2018). As a result, pruning [e.g., using PLINK (Purcell et al., 2007)] has to be employed to keep less dependent SNPs in their analysis, which inevitably leads to the loss of useful information included in correlated SNPs. Compared with the traditional single SNP analysis which only considers only one SNP each time and often suffers from power reduction (Zeng et al., 2015), the gene-based association study is another popular supplementary analysis, which examines the joint significance of a group of SNPs and has the potential to aggregate weak association signals across multiple genetic variants and is thus more powerful (Zeng et al., 2014). Moreover, gene-based associations are easily to interpret because gene is a more meaningfully biological unit compared with individual genetic variant.

**FIGURE 1:**
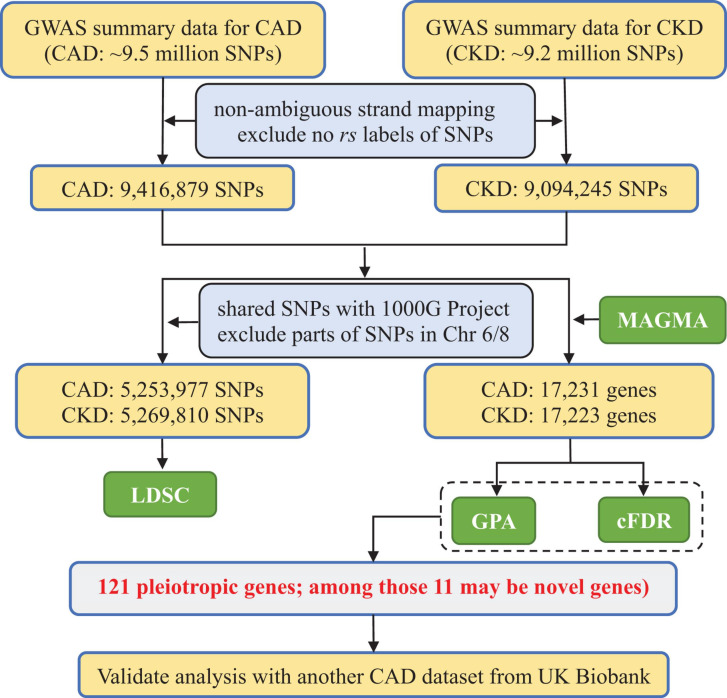
Flowchart of data preparation and analysis for CKD and CAD in the present study. CAD, coronary artery disease; CKD, chronic kidney disease; MAGMA, Multi-marker Analysis of GenoMic Annotation; LDSC, linkage disequilibrium score regression; GPA, Genetic analysis incorporating Pleiotropy and Annotation; pleiotropy-informed methods, GPA and cFDR; cFDR, conditional false discovery rate; 1000G, 1000 Genomes Project phase III.

Given the potential pleiotropy between CAD and CKD that was widely implied in previous work (Go et al., 2004; Liu et al., 2012; Ene-Iordache et al., 2016), we hypothesize that shared genes identified by different pleiotropy-informed methods should have a higher probability to be candidate pleiotropic genes. To do so, in the present study we first evaluated the overall genetic correlation between CAD and CKD with summary statistics available from large scale GWASs through cross-trait LDSC (linkage disequilibrium score regression) (Bulik-Sullivan B. et al., 2015). We next conducted a gene-based association analysis using MAGMA (Multi-marker Analysis of GenoMic Annotation) (de Leeuw et al., 2015) to integrate association signals from SNP level into gene level. We thus obtained *P*-value for each protein coding gene. Depending on those gene-level *P*-values, we detected pleiotropic genes with two pleiotropy-informed association methods including cFDR (conditional false discovery rate) (Andreassen et al., 2013; Smeland et al., 2020) and GPA (Genetic analysis incorporating Pleiotropy and Annotation) (Chung et al., 2014). We also attempted to validate our results in another CAD dataset available from the UK Biobank (UKB) cohort. The framework of our data analysis is demonstrated in Figure 1.

## Materials and Methods

### GWAS Summary Statistics

We obtained summary statistics (e.g., effect allele, effect size, and *P*-values) for CKD from the latest GWAS of the CKDGen consortium (Wuttke et al., 2019). In this study the creatinine value obtained with a Jaffé assay before 2009 was calibrated by multiplying by 0.95, and glomerular filtration rate (GFR) was estimated with the Chronic Kidney Disease Epidemiology Collaboration (CKD-EPI) equation for adults (larger than 18 years age) while using the Schwartz formula for individuals less than 18 years old and was winsorized at 15–200 ml min^–1^ per 1.73 m^2^. CKD was defined as an eGFR below 60 ml min^–1^ per 1.73 m^2^. After stringent quality control, a total of 567,460 (64,164 cases and 502,296 controls; *N*_*eff*_ = 227,584) individuals of European ancestry and ∼9.6 million SNPs for CKD were left. We yielded summary statistics of CAD from the CARDIoGRAMplusC4D Consortium (Nikpay et al., 2015), which included 184,305 (60,801 cases and 123,504 controls; *N*_*eff*_ = 162,972) individuals of European ancestry and ∼9.4 million SNPs after quality control.

We further validated our results using another summary statistic of CAD obtained from the UKB cohort^1^. The UKB-CAD dataset included 405,940 individuals of European ancestry (23,888 cases and 382,052 controls; *N*_*eff*_ = 89,929) and 23,861,747 SNPs after quality control (i.e., INFO scores >0.8, allele count at least 20 and minor allele count less than 20). The association in the UKB-CAD dataset was analyzed through the SAIGE method (Zhou et al., 2018), which implemented the logistic mixed model with a kinship matrix as random effects and age, sex, age × sex, age^2^, age^2^ × sex as well as the first ten principal components as fixed-effects covariates.

### Estimated Overall Genetic Correlation With LDSC

We applied the cross-trait LDSC (Bulik-Sullivan B. et al., 2015) to assess the overall genetic correlation *r*_*g*_ between CKD and CAD using all available SNPs. The software of LDSC (version v1.0.1) was downloaded at https://github.com/bulik/ldsc and our analysis was conducted with default settings. Following prior studies (Bulik-Sullivan B. et al., 2015), we performed stringent quality control procedures during the LDSC analysis: (1) excluded non-biallelic SNPs and those with strand-ambiguous alleles; (2) excluded duplicated SNPs and those having no rs labels; (3) excluded SNPs that were located within two genetic regions including major histocompatibility complex (chr6: 28,500,000–33,500,000) (Bulik-Sullivan B. et al., 2015) and chr8: 7,250,000–12,500,000 (Price et al., 2008); (4) kept SNPs that were included in the 1000 Genomes Project phase III; (5) removed SNPs whose allele did not match that in the 1000 Genomes Project phase III (The 1000 Genomes Project Consortium, 2015).

The LD scores *ℓ*_*j*_ were computed using genotypes of 7,120,251 common SNPs (minor allele frequency >0.01 and the *P*-value of Hardy Weinberg equilibrium test >1E-5) with a 10 Mb window on 503 European individuals in the 1000 Genomes Project phase III (The 1000 Genomes Project Consortium, 2015); and then regressed on the product of *Z*-score statistics of the two diseases

(1) $$E\,\left(z_{1\,j}\,z_{2\,j}\right)=\frac{\sqrt{N_{1}\,N_{2}}\,\ell_{j}}{M}\times r_{g}+\frac{\rho \,N_{s}}{\sqrt{N_{1}\,N_{2}}}$$

where *N*_1_ and *N*_2_ are the sample sizes for CAD and CKD, respectively; *N*_*s*_ is the number of individuals shared by the two GWASs, and ρ is the disease correlation among the *N*_*s*_ overlapping individuals. Theoretically, SNPs with high LD will have higher χ^2^ statistics on average than those with low LD provided that the disease has a polygenic genetic foundation (Bulik-Sullivan B. K. et al., 2015). In terms of LSDC shown in (1), the regression slope provides an unbiased estimate for genetic correlation *r*_*g*_ and is in general not influenced by sample overlap (Bulik-Sullivan B. et al., 2015).

### Summary Statistics-Based Gene-Level Association With MAGMA

Many gene-based association approaches with only summary statistics have been developed recently; among those MAGMA is a fast and flexible method and widely employed (de Leeuw et al., 2015). During the implementation of MAGMA, we defined the set of SNPs that were located within a given gene in terms of the annotation file provided in VAGIS (Liu et al., 2010). For numerical stability, we only focused on protein coding genes with at least ten SNPs (note that, this threshold was to some extent chosen arbitrarily). The genotypes of 503 European individuals in the 1000 Genomes Project phase III (The 1000 Genomes Project Consortium, 2015) were exploited as reference panel for calculating the LD matrix to incorporate the correlation structure among SNPs. After the implementation of MAGMA, the *P*-value for each gene can be available in the CAD or CKD GWAS. Depending on those *P*-values we attempted to discover significant genes that were related to CAD or CKD as well as potentially pleiotropic genes that were associated with both the two types of disease. To detect newly novel association signals, we ruled out identified genes located within 1 Mb on each side of previously reported CAD- or CKD associated genes or SNPs from the GWAS Catalog^2^ as done similarly in other studies (Bis et al., 2020). Of note, doing this was a conservative strategy and might miss potentially important association signals although false discoveries were well controlled.

### Pleiotropy-Informed Association Methods With Summary Statistics

To further leverage the pleiotropic information shared between CAD and CKD to identify gene association signals more efficiently, we employed two novel statistical genetic methods in the following. First, we utilized the cFDR method (Andreassen et al., 2013) which extended the unconditional FDR (Benjamini et al., 2001) from an empirical Bayes perspective. The cFDR measures the probability of the association of the principal disease conditioned on the strength of association with the conditional disease (Andreassen et al., 2013)

(2) $$\text{cFDR}\left(p_{i}||P_{i}\leq p_{i},P_{j}\leq p_{j}\right)$$

where *p*_*i*_ and *p*_*j*_ are the observed *P*-values of a particular gene of the principal and conditional diseases, respectively; $H_{0}^{\left(i\right)}$ denotes the null hypothesis that there does not exist association between the gene and the principal disease.

Besides cFDR, we also carried out the GPA analysis (Chung et al., 2014), which was constructed as

π=00Prob(Z=j⁢001):(P|j⁢1Z=j⁢001)∼

U[0,1],(P|j⁢2Zj⁢00=1)∼U[0,1]

π=10Prob(Z=j⁢101):(P|j⁢1Z=j⁢101)∼

Beta(α,11),(P|j⁢2Z=j⁢101)∼U[0,1]

π=01Prob(Z=j⁢011):(P|j⁢1Z=j⁢011)∼

U[0,1],(P|j⁢2Zj⁢01=1)∼Beta(α,21)

π=11Prob(Z=j⁢111):(P|j⁢1Z=j⁢111)∼

(3) Beta(α,11),(P|j⁢2Z=j⁢111)∼Beta(α,21)

where the latent variables Z*_*j*_* = (Z*_*j*_*_00_, Z*_*j*_*_10_, Z*_*j*_*_01_, Z*_*j*_*_11_) indicates the association between the *j*-th gene and the two diseases: Z*_*j*_*_00_ = 1 denotes the *j*-th gene is associated with neither of them (with probability π_00_), Z*_*j*_*_10_ = 1 denotes the *j*-th gene is only associated with the first one (with probability π_10_), Z*_*j*_*_01_ = 1 denotes the *j*-th gene is only associated with the second one (with probability π_01_), and Z*_*j*_*_11_ = 1 denotes the *j*-th gene is associated with both the diseases (with probability π_11_), indicating the extent of common biological pathways to which the two diseases may share (Chung et al., 2014). In addition, α_1_ and α_2_ (0 < α*_*k*_* < 1, *k* = 1, 2) are unknown shape parameters of the Beta distribution.

### Functional Analysis

To explore functional features of newly discovered pleiotropic genes, we performed functional enrichment analysis [e.g., Gene Ontology (GO) and KEGG pathway analysis] with DAVID 6.8^3^ (Huang da et al., 2009). Enrichment analysis allows us to validate our findings by determining functional annotations for those genes with pleiotropic effects. We also conducted the protein–protein interaction analysis to detect interaction and association in terms of the Search Tool for the Retrieval of Interacting Genes/Proteins (STRING 11.0 at https://string-db.org/) database (Szklarczyk et al., 2019). We implemented the signaling pathways of these significant genes by Cytoscape software and visualized them by CluePedia (Bindea et al., 2009).

## Results

### Estimated Overall Genetic Correlation Between CAD and CKD

After quality control, a total of 5,253,977 and 5,269,810 genetic variants are reserved for CAD or CKD, respectively. The genome-wide SNP-based heritability is estimated to be 4.69% (SE = 0.35%) for CAD and 0.53% (SE = 0.12%) for CKD with LDSC. The genomic inflation factor (i.e., the ratio of the observed median χ^2^ statistic to the expected median) is 1.015 for CAD and 1.143 for CKD, which, together the LDSC intercept [i.e., 0.903 (SE = 0.005) for CAD and 1.134 (SE = 0.007) for CKD], suggests that the weak inflation of the χ^2^ statistic of CKD is primarily due to polygenicity rather than population stratification or cryptic relatedness. In terms of those results, the adjustment of genomic control is also not necessary.

**FIGURE 2:**
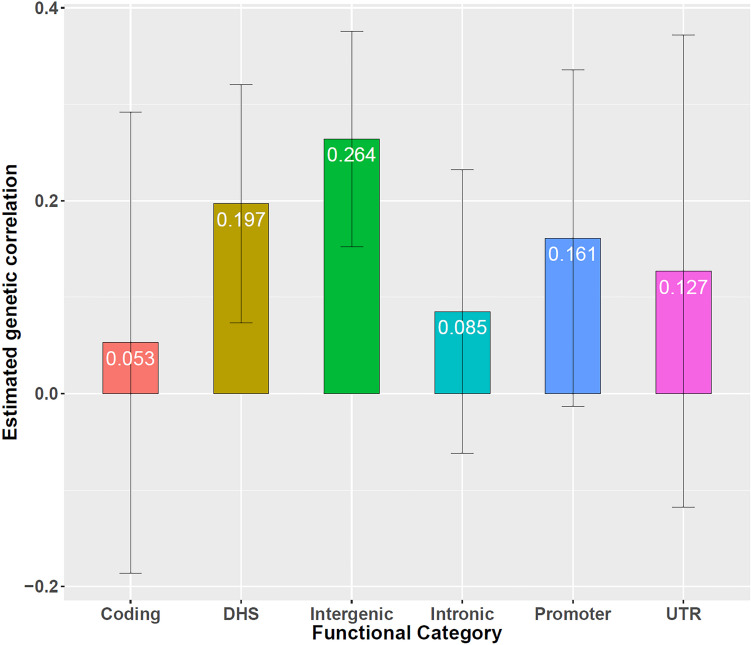
Genetic correlation between CAD and CKD in six functional categories, including coding, UTR, promoter, DHS, intronic, and intergenic. Error bars show 1.96 × SE. Besides DHS and intergenic, the genetic correlation is ${\hat{r}}_{g}=0.053$ (SE = 0.122, *P* = 6.64E-1) for coding, ${\hat{r}}_{g}=0.127$ (SE = 0.125, *P* = 3.10E-1) for UTR, ${\hat{r}}_{g}=0.161$ (SE = 0.089, *P* = 7.00E-2) for promoter, ${\hat{r}}_{g}=0.089$ (SE = 0.075, *P* = 2.55E-1) for intronic.

Next, based on all overlapped genetic variants (i.e., 5,117,020 SNPs), using LDSC we observe there exists a positive genetic correlation between the two types of diseases [${\hat{r}}_{g}=0.173$, 95% confidence interval (CI) 0.023 ∼ 0.332, *P* = 0.024], providing empirical evidence that the two diseases share common genetic components. We further quantify genetic correlation between CAD and CKD separately in six functional categories (Gusev et al., 2014), including coding, UTR (untranslated region), promoter, DHS (DNaseI hypersensitivity sites), intronic and intergenic. It is found that all the estimates of *r*_*g*_ in those categories are positive, again supporting the statement that CAD and CKD have overlapped genetic foundation. In particular, there exists a significantly positive genetic correlation in the regions of DHS (${\hat{r}}_{g}=0.197$, 95% CI 0.074 ∼ 0.319, *P* = 1.60E-3) and intergenic (${\hat{r}}_{g}=0.264$, 95% CI 0.153 ∼ 0.375, *P* = 3.03E-6) (Supplementary Table S2 and Figure 2).

Overall, through genetic correlation analysis we reveal that CAD and CKD are genetically similar and share moderate overlap in genetic etiology, especially at some certain regions. Therefore, it is worthy of additional investigation into shared genetic mechanisms through pleiotropy-informed statistical tools.

### Associated Genes Identified With MAGMA, cFDR, and GPA

In our gene-based association analysis, we assign a set of genetic variants to predefined genes and obtain a total of 17,231 and 17,223 protein coding genes for CAD or CKD, respectively. Using MAGMA, we identify 763 CAD-associated genes and 827 CKD-associated genes (FDR < 0.05) (Supplementary Tables 3, 4 and Supplementary Figure 1). Importantly, 25.8% (=197/763) CAD-associated genes (e.g., *ACER2*, *ACSS2*, *ARHGEF19*, and *BBS10*) and 60.7% (=503/827) CKD-associated genes (e.g., *BAG6*, *BAK1*, *BTNL2*, and *C4BPB*) are likely novel genes because those genes are not nearby (within 1 Mb upstream and downstream) any previous GWAS index SNPs or associated genes in terms of the GWAS catalog (McMahon et al., 2019).

In our cFDR analysis the Q–Q plot of CAD conditional on the nominal *P*-value of CKD illustrates the existence of enrichment at different significance thresholds of CKD (Supplementary Figure 2A). The presence of leftward shift suggests that the proportion of true associations for a given CKD *P*-value would increase when the analysis is limited to include more significant SNPs. On the other hand, in terms of the Q–Q plot of CKD conditional on the nominal *P*-value of CAD (Supplementary Figure 2B), we observe a more pronounced separation in different curves, implying that there exists a stronger enrichment for CKD given CAD than that for CAD given CKD. We further formally analyze the two diseases jointly using cFDR and show the results in Supplementary Tables 5, 6 and Supplementary Figure 3. Briefly, with cFDR we identify 875 CAD-associated genes and 1,062 CKD-associated genes (cFDR < 0.05). Among those genes, 243 CAD-associated and 639 CKD-associated genes are possibly novel (Supplementary Tables 5, 6). More interesting, all CAD-associated genes identified by MAGMA are replicated and 111 additional genes are discovered (Supplementary Figure 4); and all CKD-associated genes identified by MAGMA are also verified and 234 more genes are newly discovered (Supplementary Figure 5).

We next employ GPA to implement another integrative analysis for the two diseases. In terms of the GPA result we discover 504 and 1395 significant genes that are related to CAD or CKD (Supplementary Tables 7, S8 and Supplementary Figure 6). Among those, 17.3% (=87/504) novel CAD-associated genes (e.g., *ACVR2A*, *AP3M1*, *ARHGEF19*, and *BACH1*) and 61.2% (=854/1395) CKD-associated genes (e.g., *ABCA4*, *ABCC2*, *ABCF3*, and *ACOX1*) may be newly novel genes because they are not nearby (within 1 Mb upstream and downstream) any previous GWAS index SNPs or associated genes in terms of the GWAS catalog (McMahon et al., 2019). Furthermore, we find 504 CAD-associated and 770 CKD-associated genes that are identified simultaneously by GPA and MAGMA (Supplementary Figures 7, 8).

### Identified Pleiotropic Gene With Both cFDR and GPA

**FIGURE 3:**
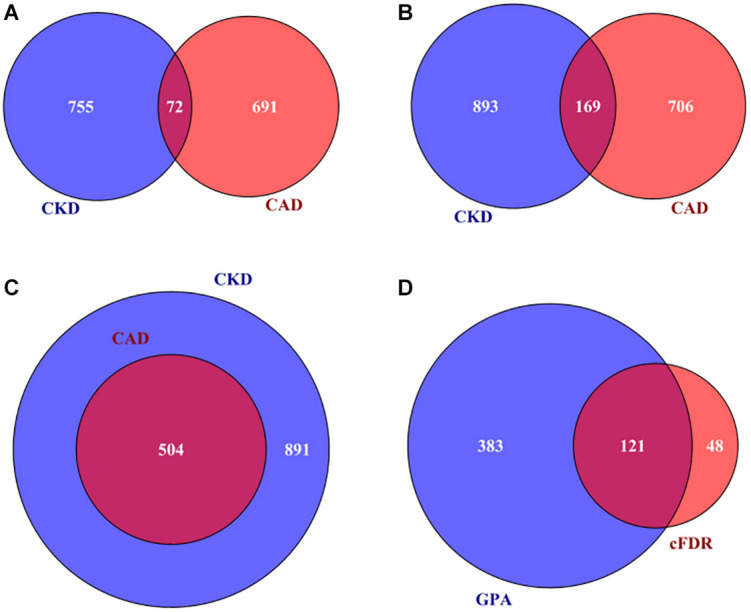
**(A)** A total of 72 associated genes shared by CAD and CKD using MAGMA; **(B)** 169 associated genes shared by CAD and CKD using cFDR; **(C)** a total of 504 genes shared by CAD and CKD using GPA; **(D)** a total of 121 pleiotropic genes of CAD and CKD simultaneously discovered by cFDR and GPA. CAD, coronary artery disease; CKD, chronic kidney disease; MAGMA, Multi-marker Analysis of GenoMic Annotation; GPA, Genetic analysis incorporating Pleiotropy and Annotation; cFDR, conditional false discovery rate.

According to the result of MAGMA, 72 genes are related to both CAD and CKD (Supplementary Table 9 and Figure 3A). Based on the two integrative analyses, 169 genes are shared between CAD and CKD when using cFDR (Supplementary Table 10 and Figure 3B) and 504 genes are shared between CAD and CKD when using GPA (Supplementary Table 11 and Figure 3C). In addition, through GPA we observe that a substantial fraction of genes that are simultaneously related to CAD and CKD, with π_11_ estimated to be 8.2% (SE = 0.1%), offering additional statistical evidence supporting the existence of pleiotropy between CAD and CKD [the statistic of the likelihood ratio test is 225.6 and *P* = 5.35E-51 (Chung et al., 2014)].

Due to the difference of power in identifying pleiotropic genes via cFDR or GPA, we expect that a gene would be more likely to have pleiotropic effect if it is discovered by cFDR and GPA simultaneously. Relying on this principle we define a set of 121 genes that are associated with CAD and CKD and are jointly detected by cFDR and GPA to be pleiotropic genes (Supplementary Table 12 and Figure 3D), among which five (i.e., *IGF2R*, *LPA*, *BCAS3*, *SLC22A2*, and *ATXN2*) were identified in previous studies (Supplementary Table 1). Furthermore, after ruling out genes located within 1 Mb on each side of previously reported genes or SNPs, we ultimately discover 11 newly novel pleiotropic genes associated with both CAD and CKD (i.e., *RHGEF19*, *RSG1*, *NDST2*, *CAMK2G*, *VCL*, *LRP10*, *RBM23*, *USP10*, *WNT9B*, *GOSR2*, and *RPRML*) (Table 1 and Supplementary Figures 9–13).

**TABLE 1:** Pleiotropic genes associated with CAD and CKD identified by cFDR and GPA jointly.

Gene	CHR	Position	cFDR		GPA	
				
			CAD	CKD	CAD	CKD
*ARHGEF19*	1	16,424,598–16,639,104	2.55E-03	4.16E-02	1.19E-02	1.76E-03
*RSG1*	1	16,458,181–16,663,659	2.83E-03	3.38E-02	1.28E-02	1.47E-03
*NDST2*	10	75,461,668–75,671,589	1.09E-02	4.13E-03	4.69E-02	8.63E-04
*CAMK2G*	10	75,472,258–75,734,349	5.06E-03	3.02E-03	2.51E-02	4.13E-04
*VCL*	10	75,657,871–75,979,914	6.07E-03	1.52E-02	2.75E-02	1.58E-03
*LRP10*	14	23,240,959–23,447,291	8.22E-03	2.21E-02	3.35E-02	2.42E-03
*RBM23*	14	23,269,853–23,488,396	7.62E-03	2.80E-02	3.13E-02	2.78E-03
*USP10*	16	84,633,554–84,913,527	6.48E-03	2.63E-04	4.08E-02	1.01E-04
*WNT9B*	17	44,828,967–45,054,437	4.20E-04	3.37E-02	1.84E-03	2.47E-04
*GOSR2*	17	44,900,485–45,118,733	8.13E-04	3.22E-02	4.08E-03	5.39E-04
*RPRML*	17	44,955,521–45,156,614	1.90E-03	2.16E-02	8.30E-03	6.50E-04

### Validation the Results in a Latest GWAS From the UK Biobank

We further validate the main results using the UKB-CAD summary statistics and show the results in Supplementary Tables 13–16. The genome-wide SNP-based heritability is estimated to be 2.42% (SE = 0.20%) for UKB-CAD with LDSC. We also do not observe a substantial inflation in the UKB-CAD summary statistics [the estimated λ = 1.178 with the intercept = 1.057 (SE = 0.005)].

According to the result of MAGMA, 184 genes are related to both UKB-CAD and CKD (Supplementary Table 13 and Supplementary Figure 14). Based on the two pleiotropy-informed integrative analyses, 373 genes are shared between UKB-CAD and CKD using cFDR (Supplementary Table 14 and Supplementary Figure 15) and 371 genes are shared between UKB-CAD and CKD using GPA (Supplementary Table 15 and Supplementary Figure 16). All the 11 pleiotropic genes described above are also analyzed here and five (i.e., *RSG1*, *LRP10*, *RBM23*, *WNT9B*, and *GOSR2*) are replicated (Supplementary Table 16).

### Functional Analyses for Pleiotropic Genes

We now undertake functional analyses for the 121 pleiotropic genes. Among these, most are located within chr 17 (20.7% = 25/121), followed by chr 1 (15.7% = 19/121) and chr 11 (12.4% = 15/121) (Supplementary Figure 17). In terms of the DAVID analysis, these genes are enriched in 34 GO terms (Supplementary Table 17). The top five candidate pathways include “dopamine transmembrane transporter activity” (*P* = 2.28E-04), “quaternary ammonium group transport” (*P* = 3.54E-04), “quaternary ammonium group transmembrane transporter activity” (*P* = 3.79E-04), “dopamine transport” (*P* = 7.38E-04), and “organic cation transmembrane transporter activity” (*P* = 7.89E-04). There pathways offer part of evidence supporting common genetic foundations between CAD and CKD. For instance, it has been shown that CKD patients had higher levels for some quaternary ammonium salts (e.g., choline) (Rennick et al., 1976), which were also risk factors for CAD (Guo et al., 2020). In our PPI analysis (Supplementary Figure 18), strong interactions are found among pleiotropic genes, such as *NDST2*, *CAMK2G*, *RASGRF1*, *IGF2R*, *SORT1*, and *TRIB1*. These genes were reported to be associated with organic cation transmembrane transporter, such as organic anion transporters oat1 and oat3, and organic cation transporters oct1 and oct2, which was also altered with chronic kidney failure in rats (Komazawa et al., 2013).

## Discussion

It has been widely observed that CAD and CKD share common pathological and clinical feature (Go et al., 2004; Liu et al., 2012; Tonelli et al., 2012; Ene-Iordache et al., 2016). However, the underlying genetic overlap between the two diseases remains unclear and a large proportion of genes related to CAD and CKD are yet discovered (Manolio et al., 2009). Large-scale GWASs undertaken for CAD and CKD offer an unprecedented opportunity to answer this question. In the present study a positive genetic correlation was found between CAD and CKD, implying genetic variants that were associated with the risk of CKD would be also related to the risk of CAD. This finding also partly explained the observed comorbidity of the two diseases (Go et al., 2004; Ene-Iordache et al., 2016).

Using existing well-established statistical approaches, we ultimately identified 11 novel pleiotropic genes shared by CAD and CKD, including *ARHGEF19*, *RSG1*, *NDST2*, *CAMK2G*, *VCL*, *LRP10*, *RBM23*, *USP10*, *WNT9B*, *GOSR2*, and *RPRML*, some of which were previously reported to play important roles in the pathogenesis of CAD or CKD (Agosti, 2002; Sivapalaratnam et al., 2012; Zanders, 2015). Furthermore, we also validated our main finding in an independent UKB-CAD dataset and replicated five genes.

Specifically, prior studies showed that *ARHGEF19* (Klarin et al., 2018) and *LRP10* (Sugiyama et al., 2000) were associated with total cholesterol and low-density lipoprotein (LDL) cholesterol, which were in turn related to CAD (Nissen et al., 2005) and CKD (Baigent et al., 2011). *RSG1* is involved in targeted membrane trafficking, and further involved in cilium biogenesis by regulating the transportation of cargo proteins to the basal body and apical tips of cilia with its protein (Agbu et al., 2018). Mice and humans with abnormal primary cilia can exhibit defects in cardiac morphogenesis, and also can cause kidney disease (Agbu et al., 2018).

*NDST2* encodes a member of the N-deacetylase/N-sulfotransferase subfamily, which has dual functions (N-deacetylation and N-sulfation) in processing heparin polymers (Humphries et al., 1998). Inactivation of *NDST2* may impact the atherosclerosis by altering the structure of monocytes/macrophages heparan sulfate (HS) (Gordts et al., 2014), while also alter the glomerular HS to impact the primary kidney diseases (Goode et al., 1995). *CAMK2G* belongs to the Ca^2+^/calmodulin-dependent protein kinase subfamily (Moyers et al., 1997). Vascular calcification correlates with the vessel stiffening and hypertension, and further increases the risk of atherosclerosis and myocardial infarction. It also exhibits a hugely elevated risk of cardiovascular mortality in CKD patients (Shanahan Catherine et al., 2011).

Vinculin (*VCL*) is a membrane-cytoskeletal protein, which associated with the linkage of integrin adhesion molecules to the actin cytoskeleton (Burridge and Feramisco, 1980), and the cell–cell and cell-matrix junctions, where it is thought to function in anchoring F-actin to the membrane (Geiger, 1979). Endothelial dysfunction caused by F-actin cytoskeleton disorder is a well-recognized instigator of cardiovascular diseases and CKD (Ding et al., 2016). *USP10* encodes a member of the ubiquitin-specific protease family of cysteine proteases (Wang et al., 2015; Lim et al., 2019). Inactivation of *USP10* can diminish Notch-induced target gene expression in endothelial cells. Importantly, tight quantitative and temporal control of Notch activity is essential for vascular development (Wang et al., 2015; Lim et al., 2019).

*WNT9B*, encodes the secreted signaling proteins (Garriock et al., 2007), is significantly associated with systolic blood pressure (Hoffmann et al., 2017), which is further related to the risk of CAD (Turner et al., 1998) and CKD (Jafar et al., 2003). *GOSR2* encodes a trafficking membrane protein which transports proteins among the medial- and trans-Golgi compartments (Bui et al., 1999). Due to its chromosomal location and trafficking function, *GOSR2* may be involved in familial essential hypertension (Boissé Lomax et al., 2013), and also was reported to be relevant to systolic blood pressure (Ehret et al., 2011) and CAD (van der Harst and Verweij, 2018).

The major strength of our work is that multiple pleiotropy-informed methods were implemented to detect pleiotropic genes by combining existing GWASs summary results without requiring individual-level datasets. Unlike previous studies (Andreassen et al., 2013; Chung et al., 2014; Zeng et al., 2018), we perform MAGMA methods to enrich a group of SNPs which may be likely associated with CAD or CKD but cannot reach genome-wide significance because of modest effects if using single marker analysis. Moreover, to minimize possible false discovery, we only reported pleiotropic genes that were simultaneously discovered by GPA and cFDR and thus were more likely to be related to both CAD and CKD. Therefore, our findings are robust.

Nevertheless, there are some limitations needed to state. First, we cannot replicate all these genes via *in vivo* and *in vitro* experiments. Second, the individuals involved in our study are of European ancestry, it is not clear whether the finding can be generalized to other populations because of ethnic diversity in genetics. Third, although empirical evidence shown above indicates that the newly identified pleiotropic genes may underlie certain aspects of the pathogenesis of CAD and CKD in a direct or indirect way, the causally biological mechanisms of those genes are still largely unclear; therefore, further studies are needed to completely delineate their functions on CAD and CKD.

## Conclusion

This study identifies common genetic architectures overlapped between CAD and CKD and will help to advance understanding of the molecular mechanisms underlying the comorbidity of the two diseases.

## Data Availability Statement

The original contributions presented in the study are included in the article/Supplementary Material, further inquiries can be directed to the corresponding author/s.

## Author Contributions

PZ and SH conceived the idea for the study. PZ, TW, and HC obtained the data. PZ and HC performed the data analyses and wrote the manuscript with the participation of all authors. PZ, JY, TW, and HC interpreted the results of the data analyses. All authors contributed to the article and approved the submitted version.

## Conflict of Interest

The authors declare that the research was conducted in the absence of any commercial or financial relationships that could be construed as a potential conflict of interest.

## Footnotes

1 https://pan.ukbb.broadinstitute.org/

2 www.ebi.ac.uk

3 https://david.ncifcrf.gov/
