# Supplementary material for: Improved Detection of Potentially Pleiotropic Genes in Coronary Artery Disease and Chronic Kidney Disease Using GWAS Summary Statistics
Source: Front Genet. 2020 Dec 3;11:592461. doi: 10.3389/fgene.2020.592461 (PMC7744760; doi:10.3389/fgene.2020.592461)
Supplement: Supplementary file 1 [file Data_Sheet_1.docx]

**Supplementary Result**


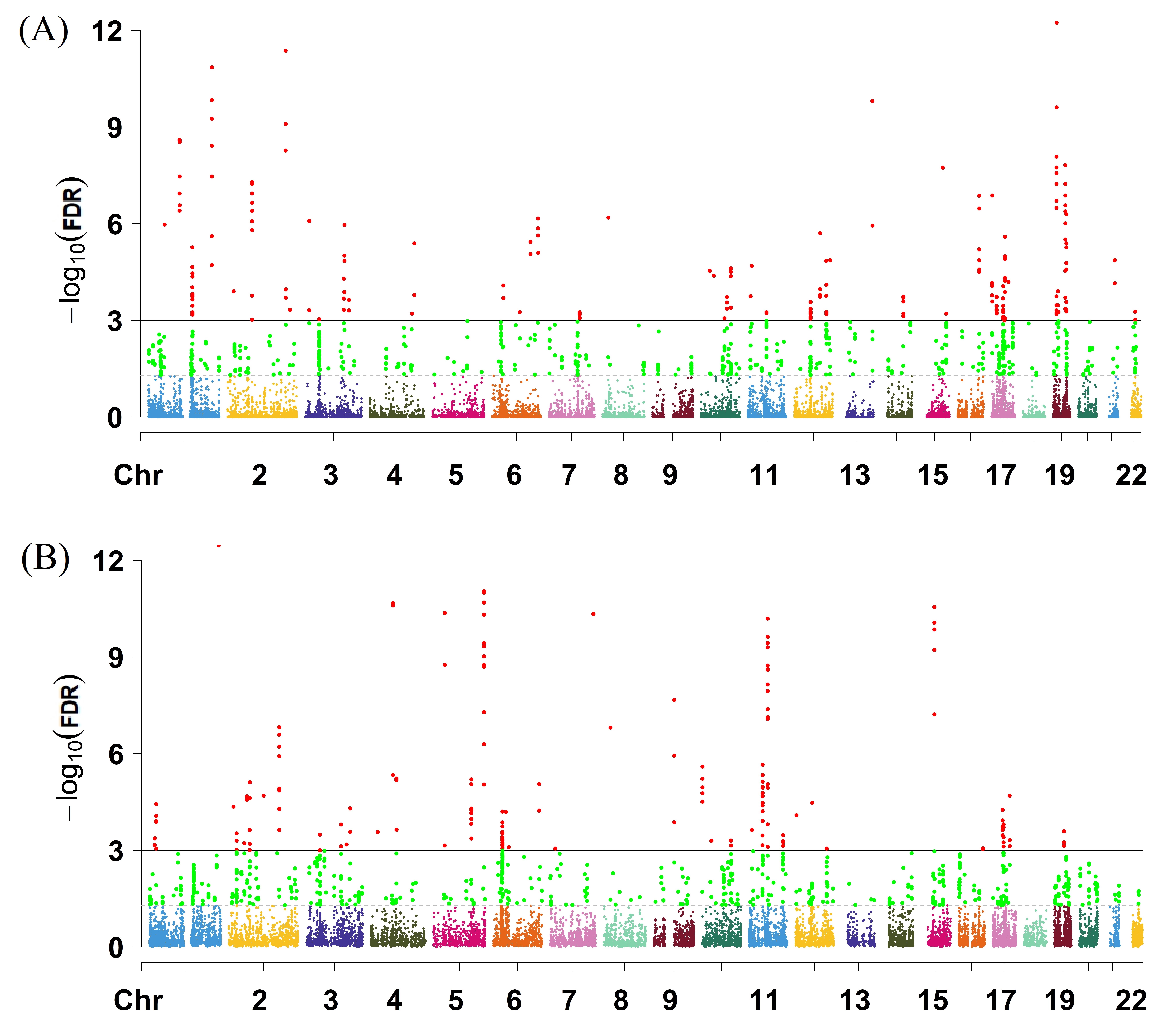


Figure S1. Manhattan plot of -log_10_FDR for (A) CAD and (B) CKD in terms of the results of MAGMA. The two horizontal reference lines in each plot indicate FDR < 0.05 or < 0.001. CAD: coronary artery disease; CKD: chronic kidney disease.


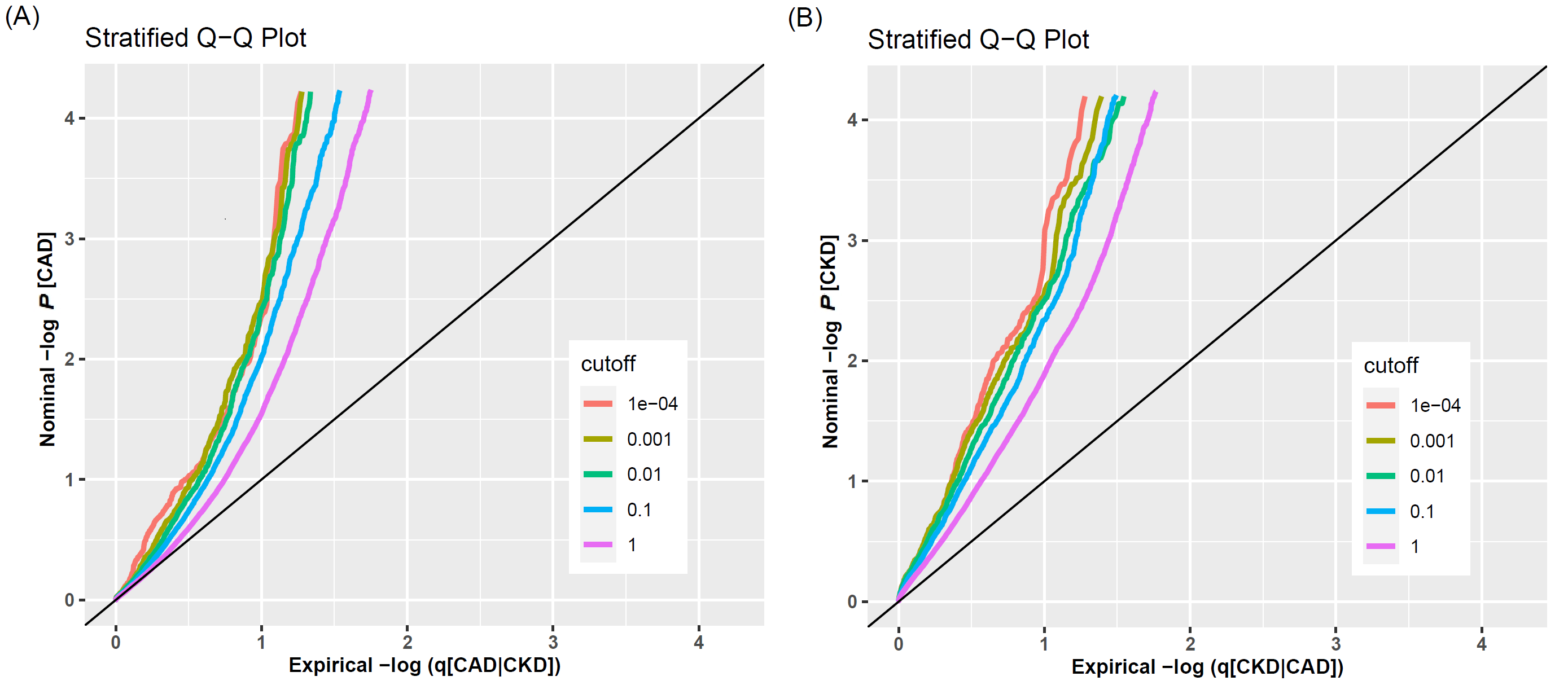


Figure S2. Conditional Q-Q plot. Stratified Q-Q plot of enrichment versus nominal -log_10_ *P*-values in (A) CAD as a function of significance of the association with CKD (CAD|CKD); (B) CKD as a function of significance of the association with CAD (CKD|CAD).


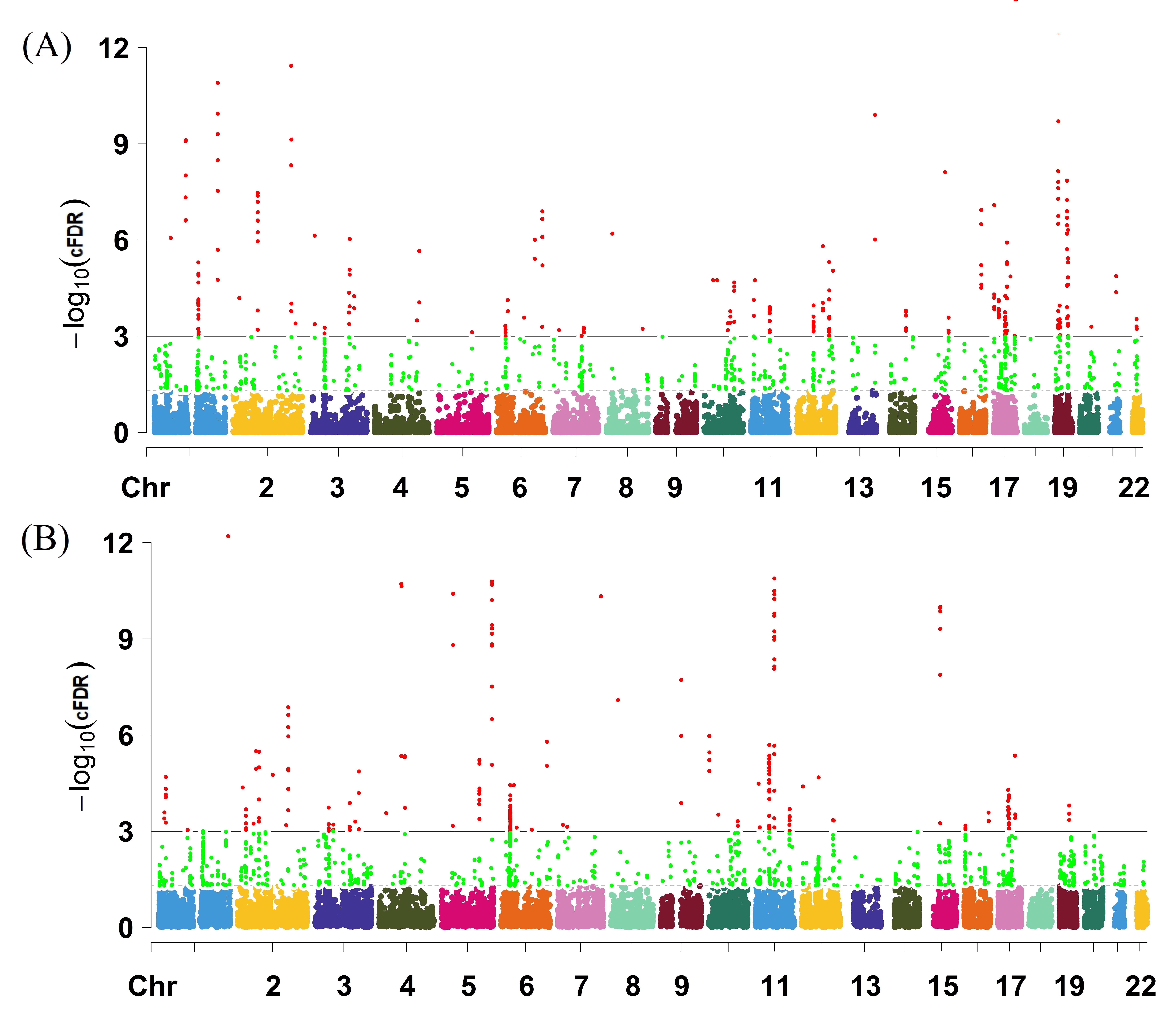


Figure S3. Manhattan plot of -log_10_FDR for (A) CAD and (B) CKD in terms of the results of cFDR. The two horizontal reference lines in each plot indicate cFDR < 0.05 or < 0.001. CAD: coronary artery disease; CKD: chronic kidney disease.


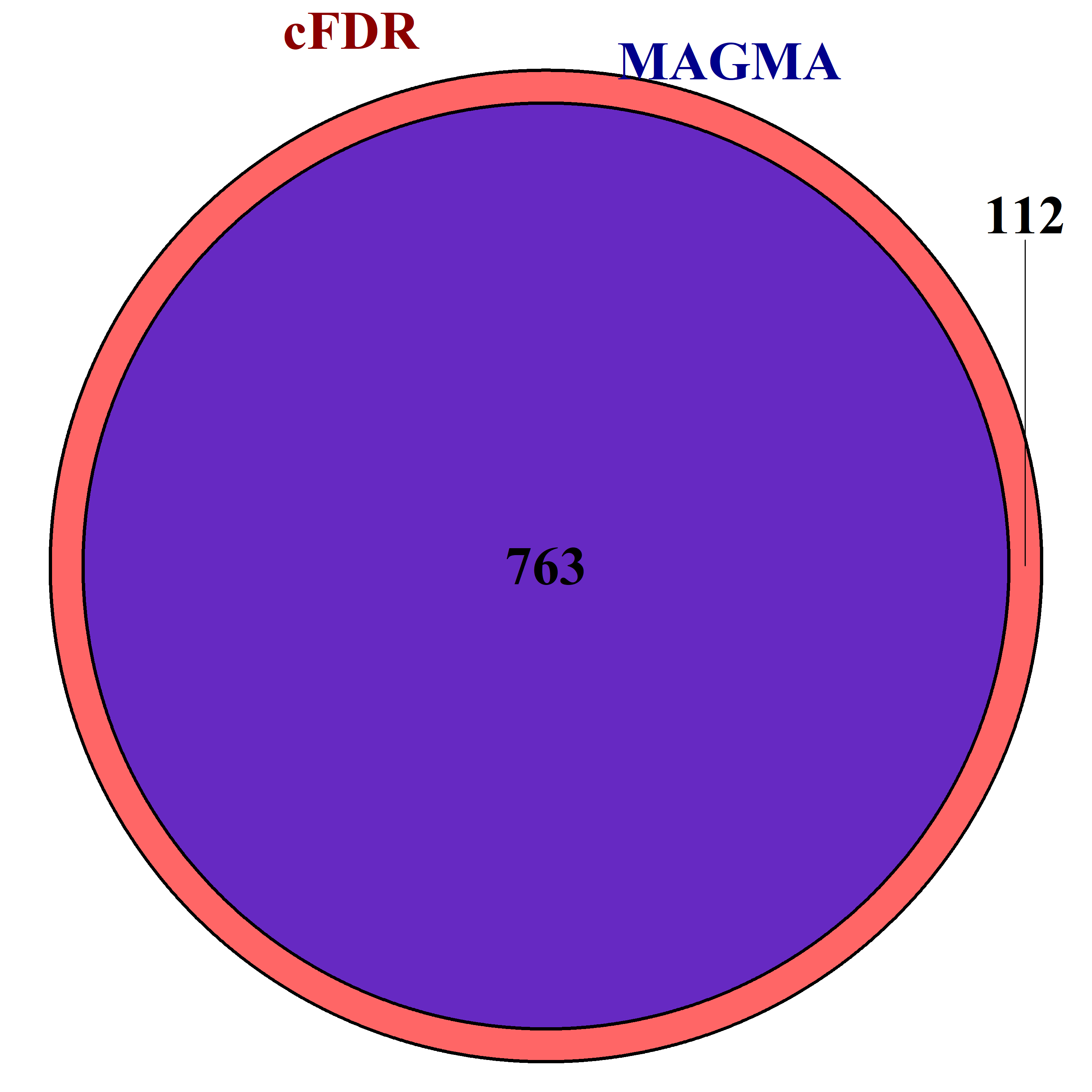


Figure S4 A total of 763 CAD-related genes simultaneously discovered by cFDR and MAGMA. CAD: coronary artery disease; MAGMA: Multi-marker Analysis of GenoMic Annotation; cFDR: conditional false discovery rate.


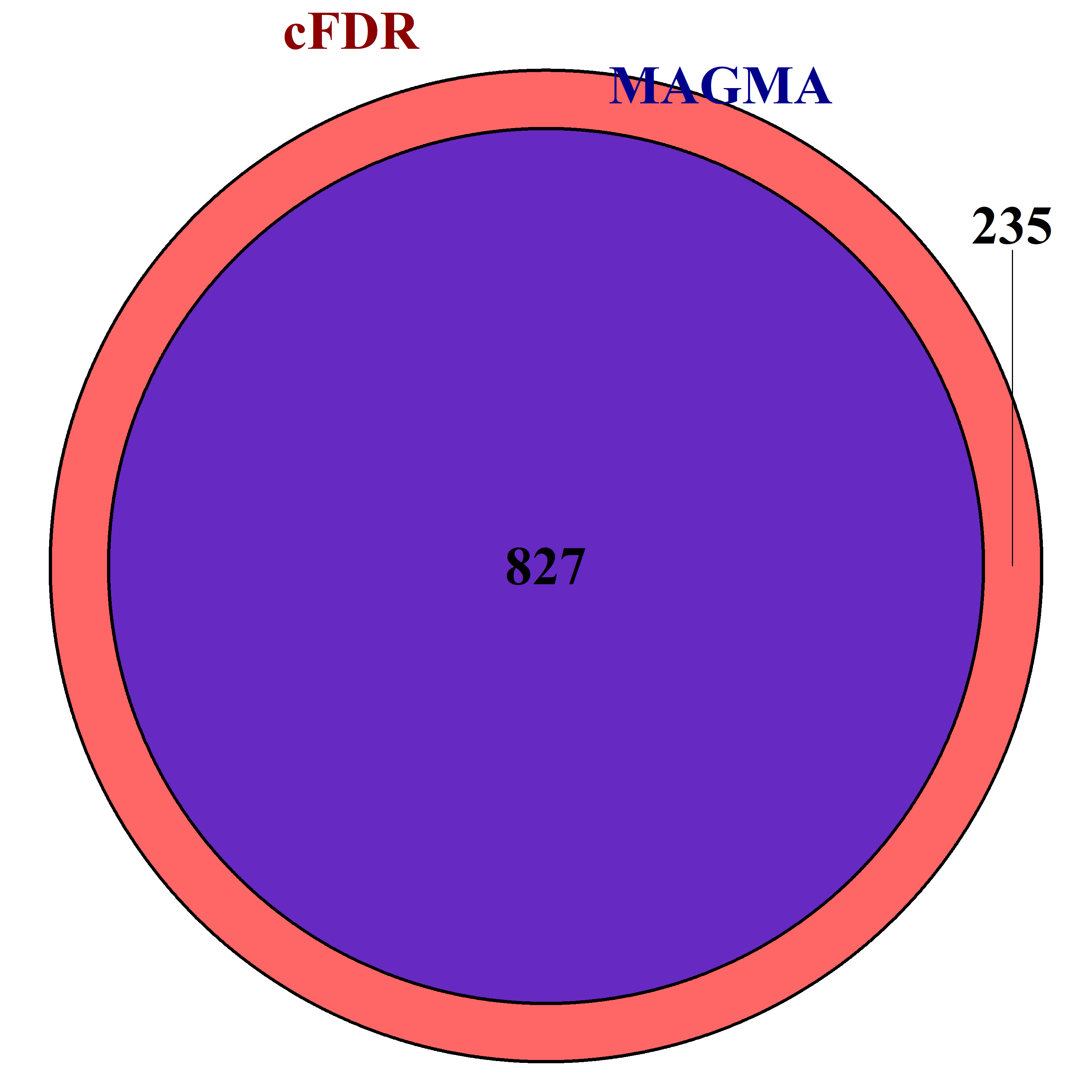


Figure S5 A total of 827 CKD-related genes simultaneously identified by cFDR and MAGMA. MAGMA: Multi-marker Analysis of GenoMic Annotation; cFDR: conditional false discovery rate; CKD: chronic kidney disease.


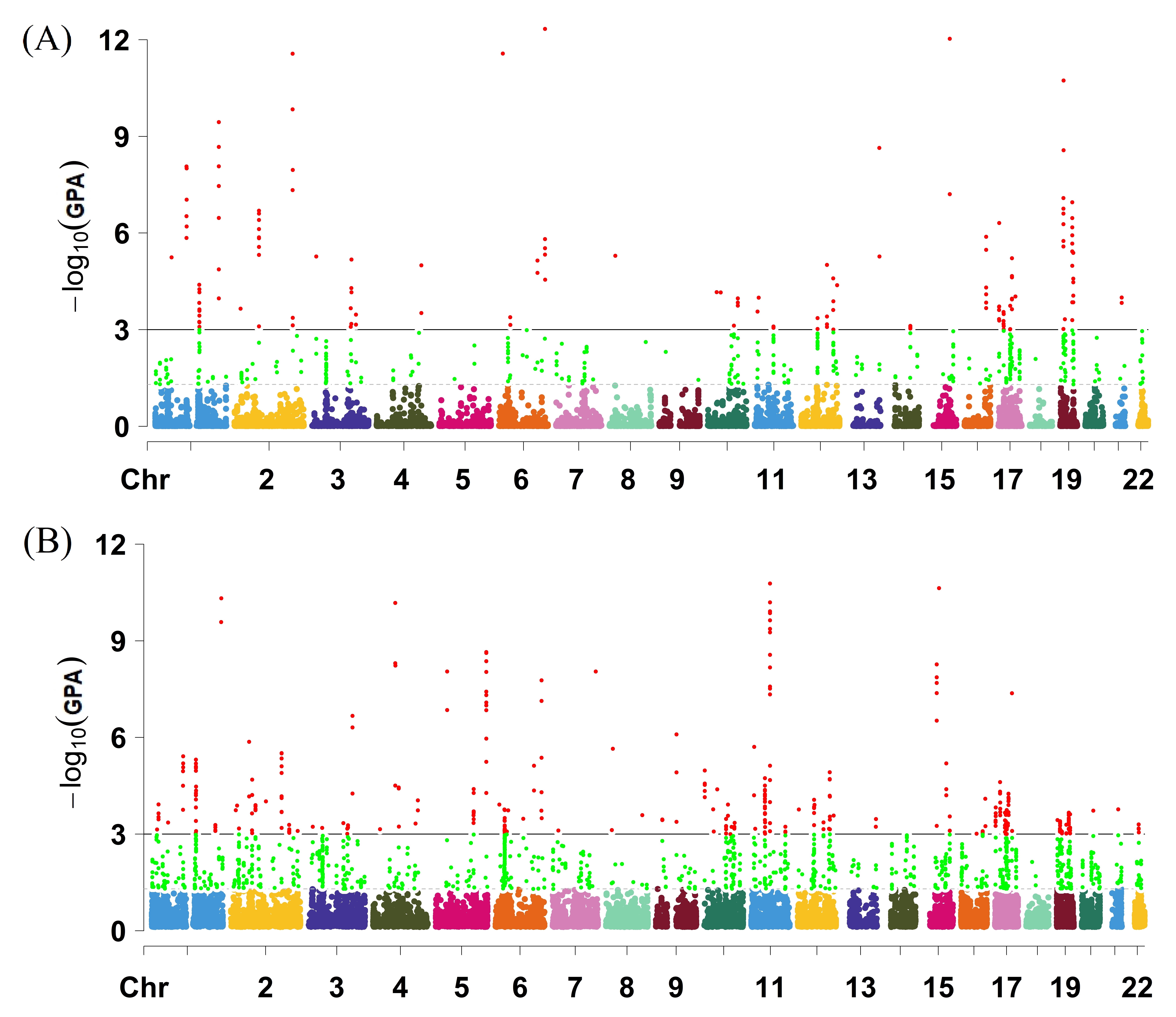


Figure S6 Manhattan plot of -log_10_FDR for (A) CAD and (B) CKD in terms of the results of GPA. The two horizontal reference lines in each plot indicate GPA < 0.05 or < 0.001. CAD: coronary artery disease; CKD: chronic kidney disease.


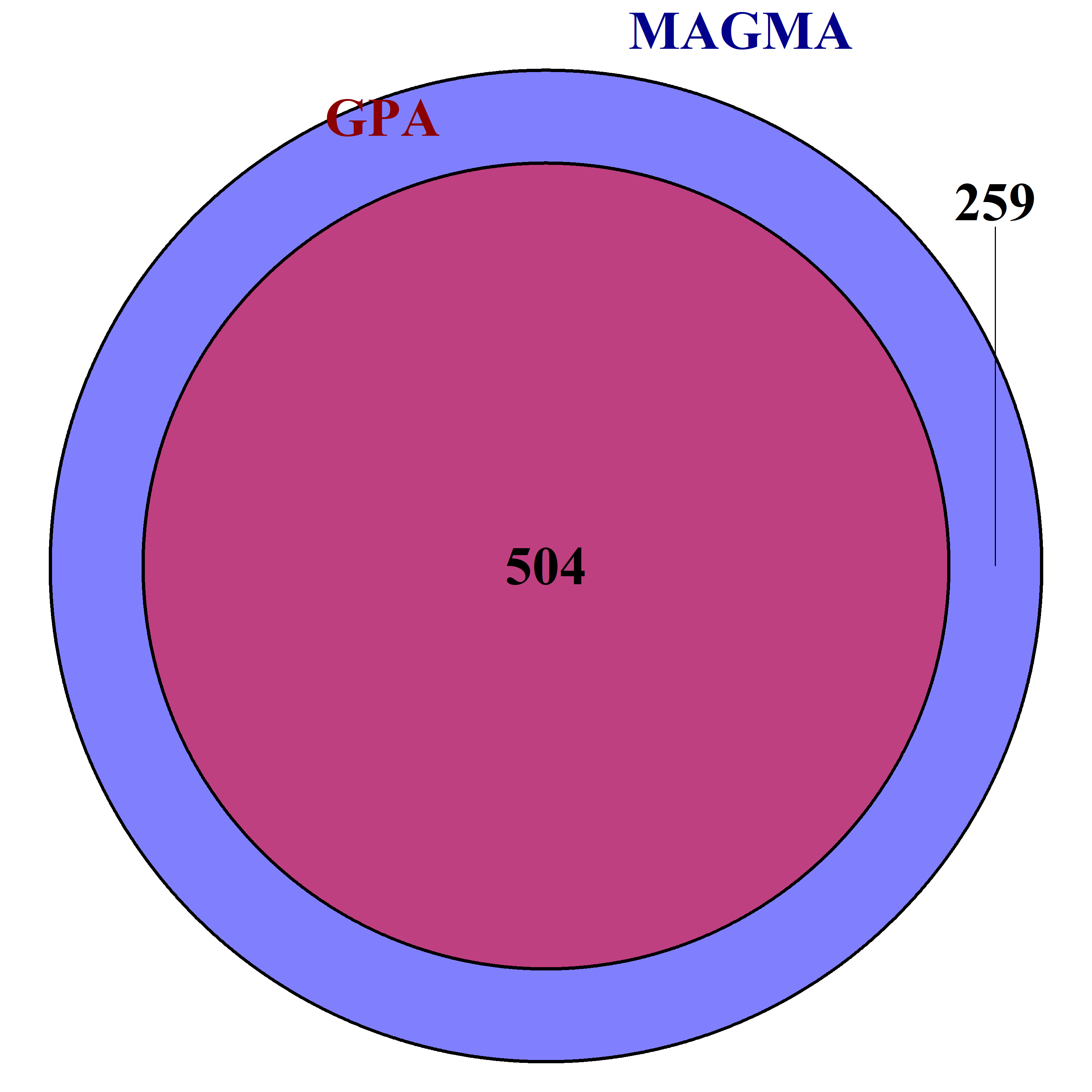


Figure S7 A total of 504 CAD-related genes simultaneously discovered by GPA and MAGMA. CAD: coronary artery disease; MAGMA: Multi-marker Analysis of GenoMic Annotation; GPA: Genetic analysis incorporating Pleiotropy and Annotation.


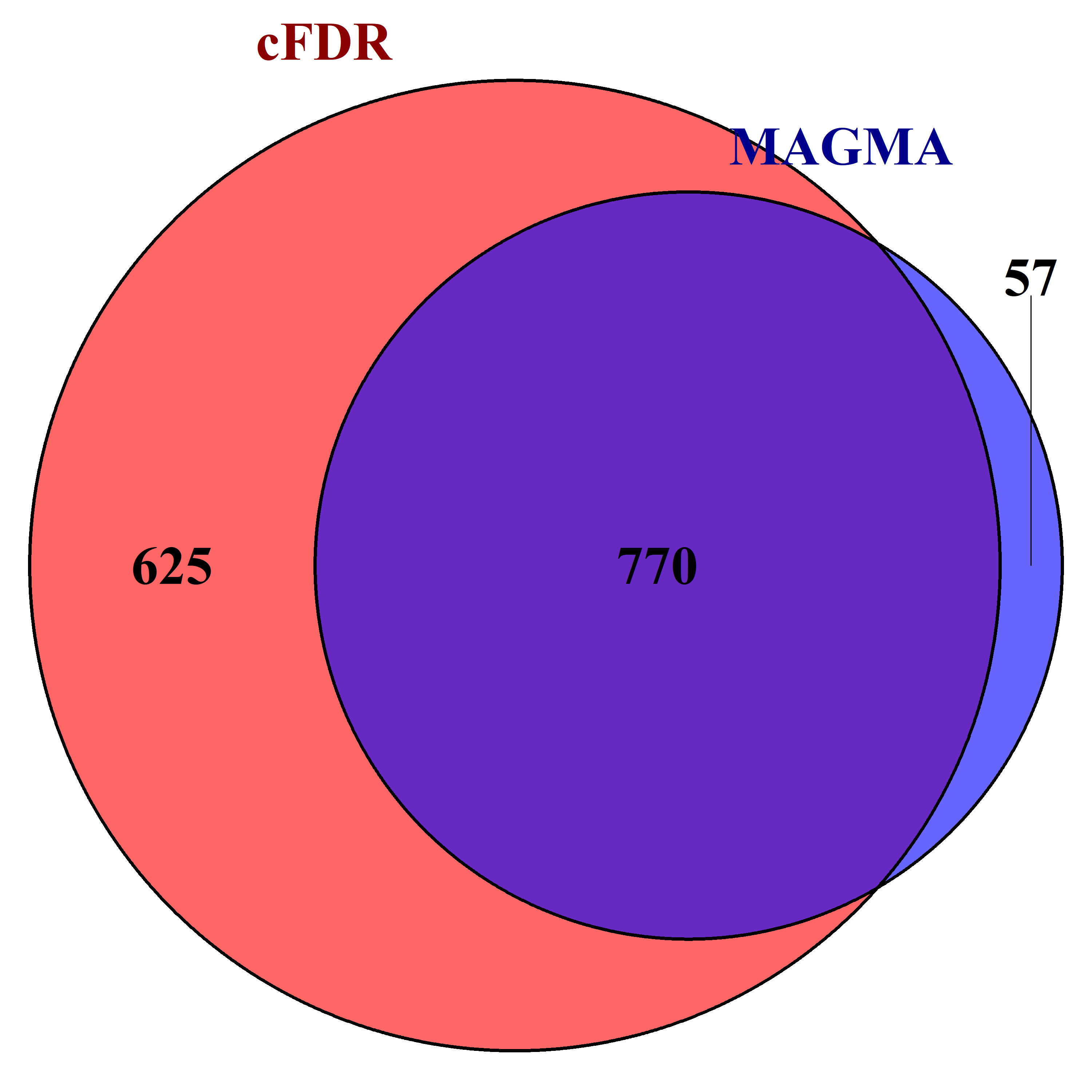


Figure S8 A total of 770 CKD-related genes simultaneously detected by GPA and MAGMA. CAD: coronary artery disease; MAGMA: Multi-marker Analysis of GenoMic Annotation; GPA: Genetic analysis incorporating Pleiotropy and Annotation.


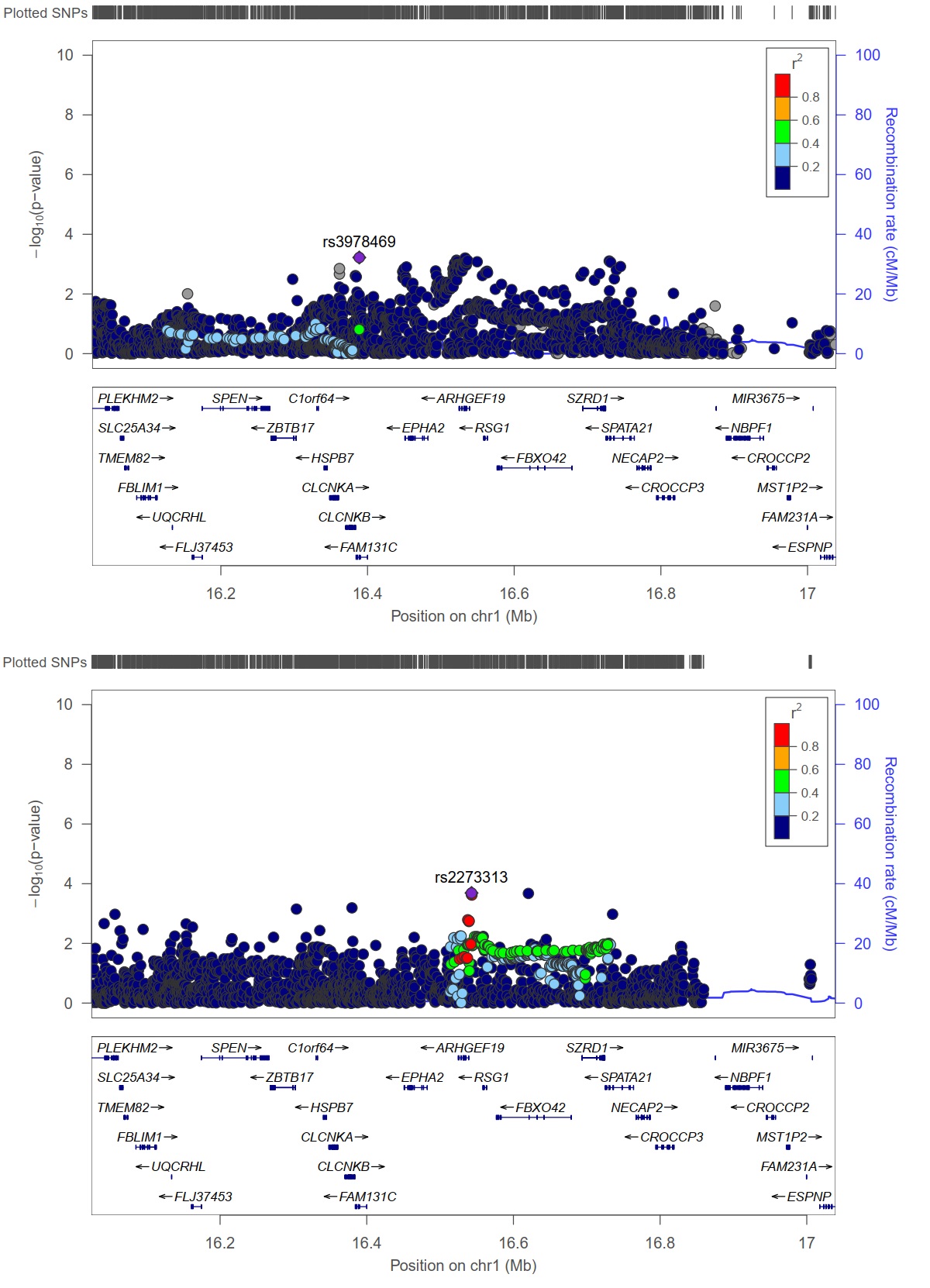


Figure S9 Top: Genetic position of *ARHGEF19* and *RSG1* for CAD; Bottom: Genetic position of *ARHGEF19* and *RSG1* for CKD.


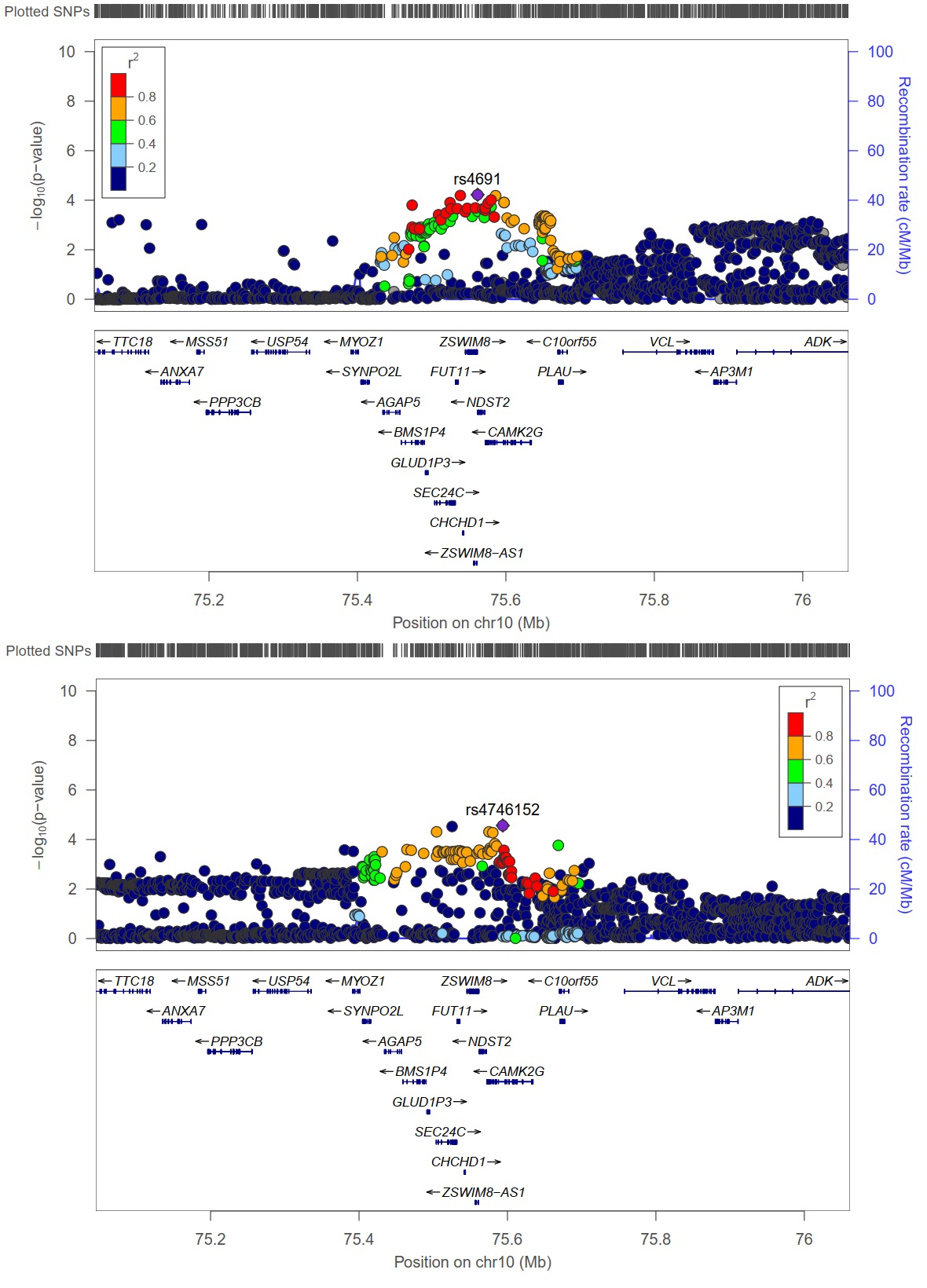


Figure S10 Top: Genetic position of *NDST2*, *CAMK2G* and *VCL* for CAD; Bottom: Genetic position of the *NDST2*, *CAMK2G* and *VCL* for CKD.


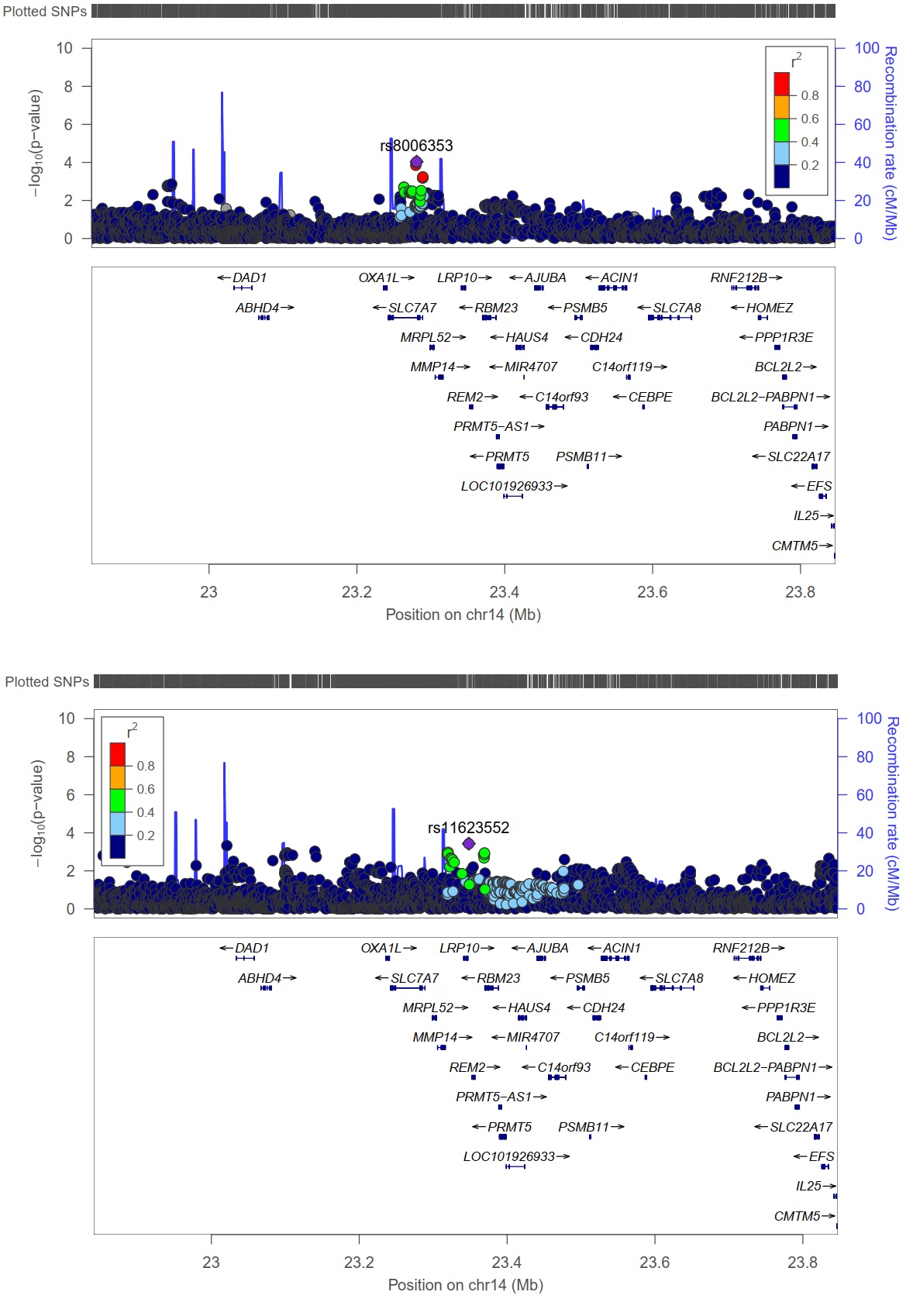


Figure S11 Top: Genetic position of *LRP10* and *RBM23* for CAD; Bottom: Genetic position of *LRP10* and *RBM23* for CKD.


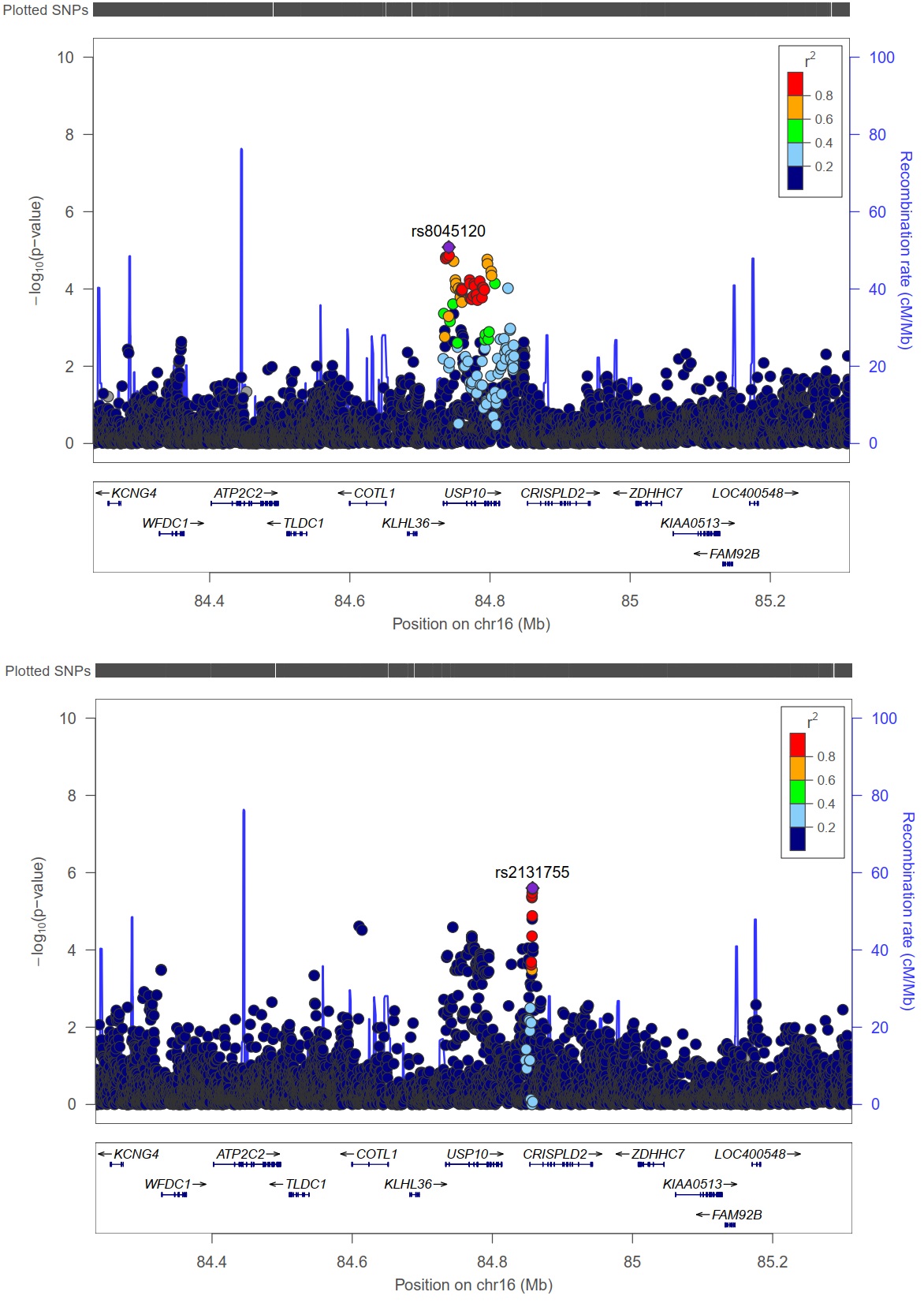


Figure S12 Top: Genetic position of *USP10* for CAD; Bottom: Genetic position of *USP10* for CKD.


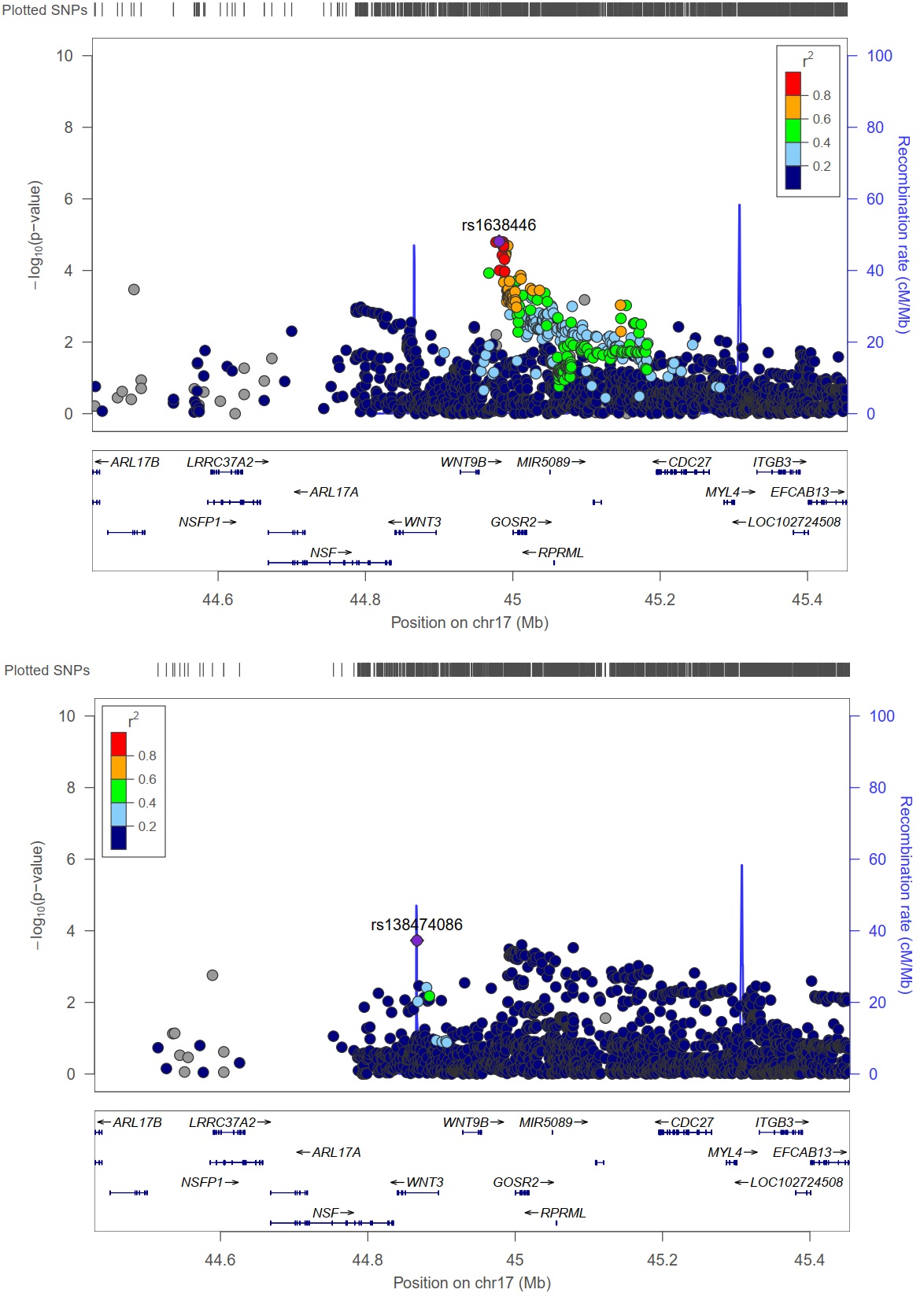


Figure S13 Top: Genetic position of *WNT9B*, *GOSR2* and *RPRML* for CAD; Bottom: Genetic position of *WNT9B*, *GOSR2* and *RPRML* for CKD.


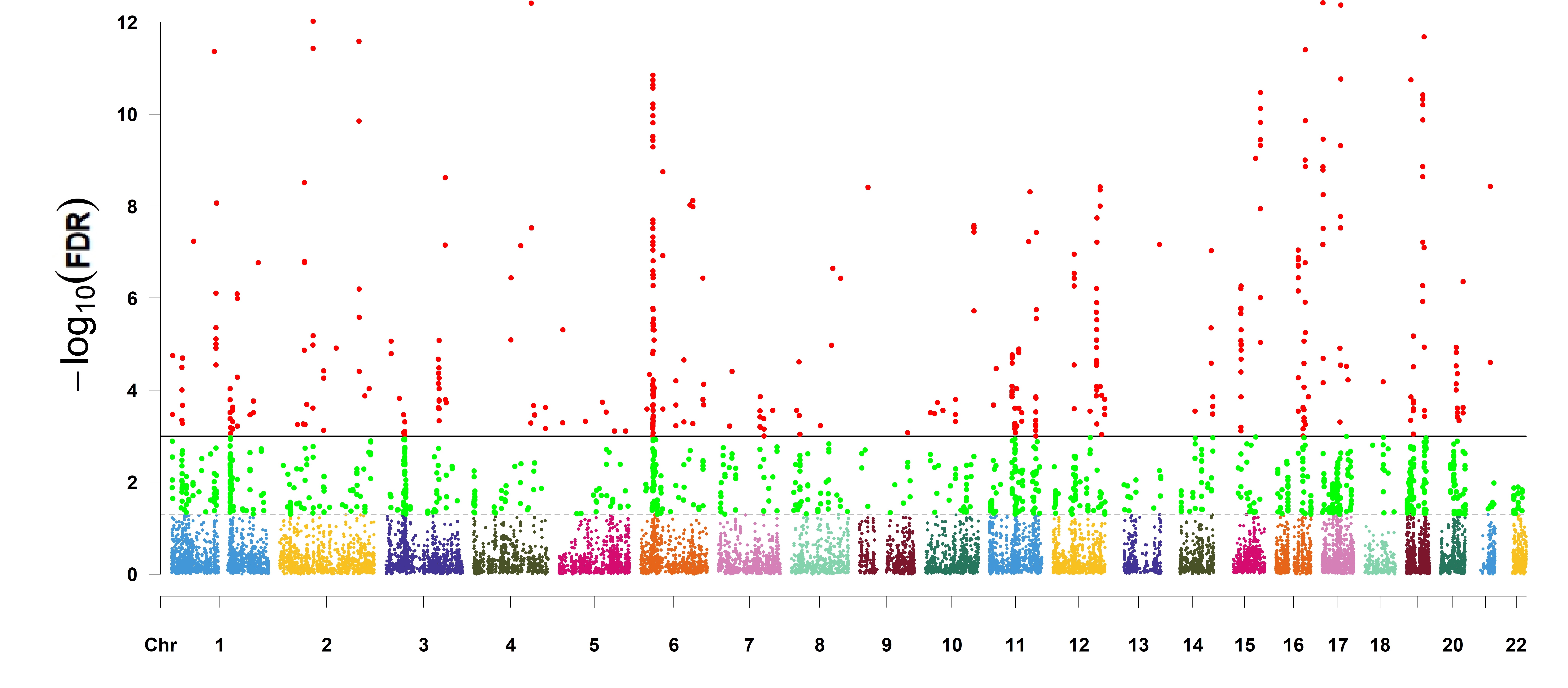


Figure S14. Manhattan plot of -log_10_FDR for UKB-CAD in terms of the results of MAGMA. The two horizontal reference lines in each plot indicate FDR < 0.05 or < 0.001. CAD: coronary artery disease.


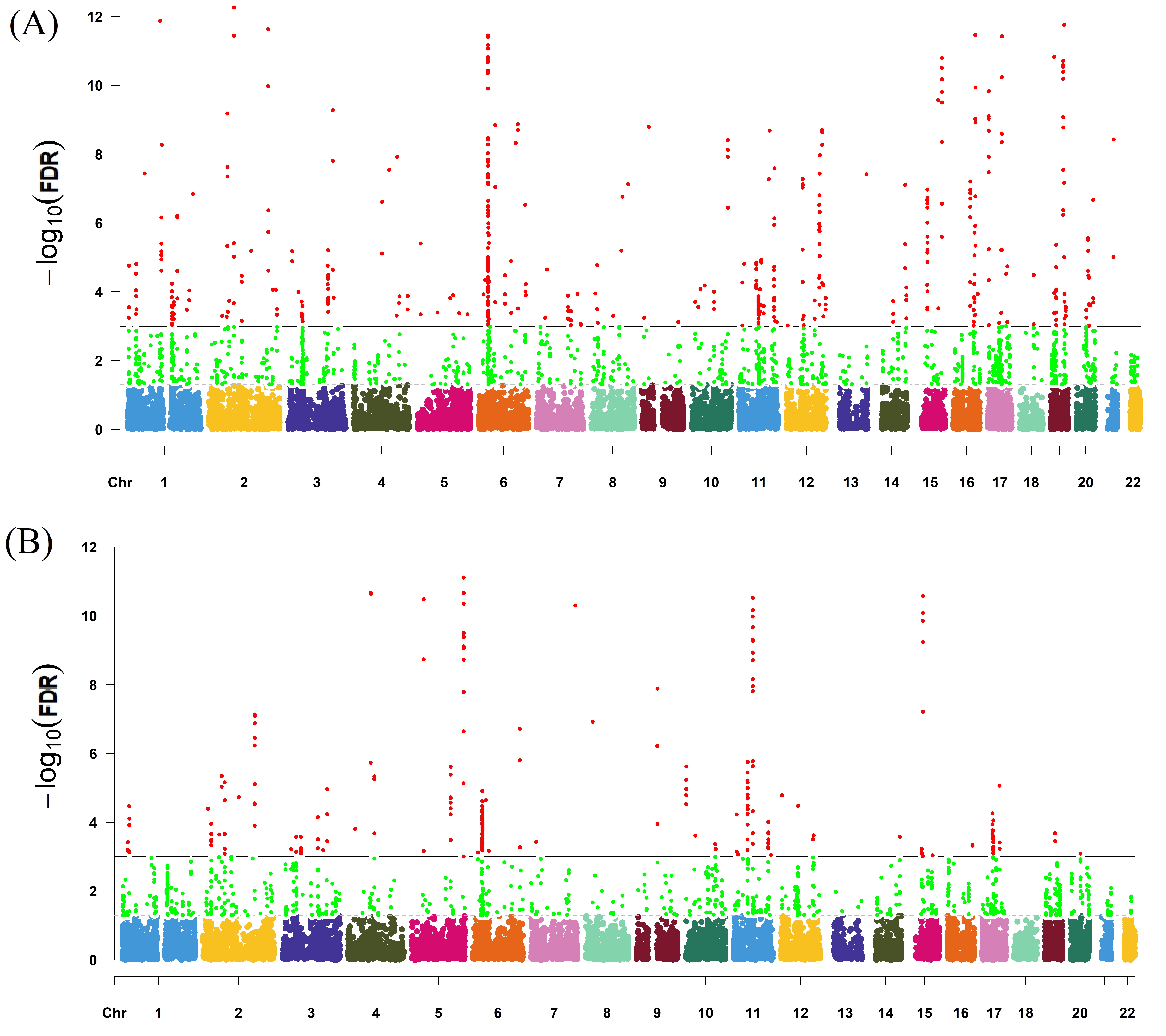


Figure S15. Manhattan plot of -log_10_FDR for (A) UKB-CAD and (B) CKD in terms of the results of cFDR. The two horizontal reference lines in each plot indicate cFDR < 0.05 or < 0.001. CAD: coronary artery disease; CKD: chronic kidney disease.


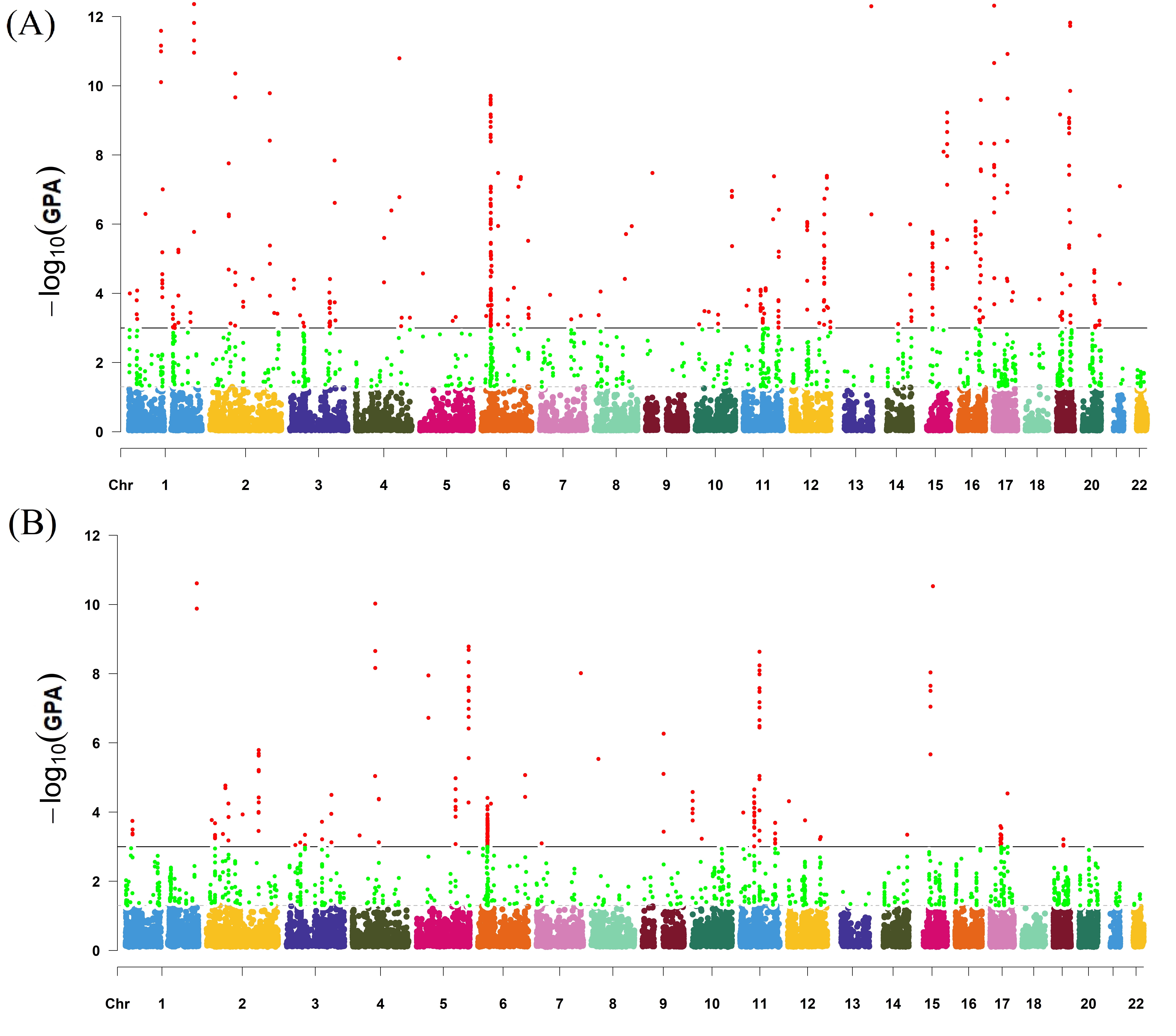


Figure S16 Manhattan plot of -log_10_FDR for (A) UKB-CAD and (B) CKD in terms of the results of GPA. The two horizontal reference lines in each plot indicate GPA < 0.05 or < 0.001. CAD: coronary artery disease; CKD: chronic kidney disease.


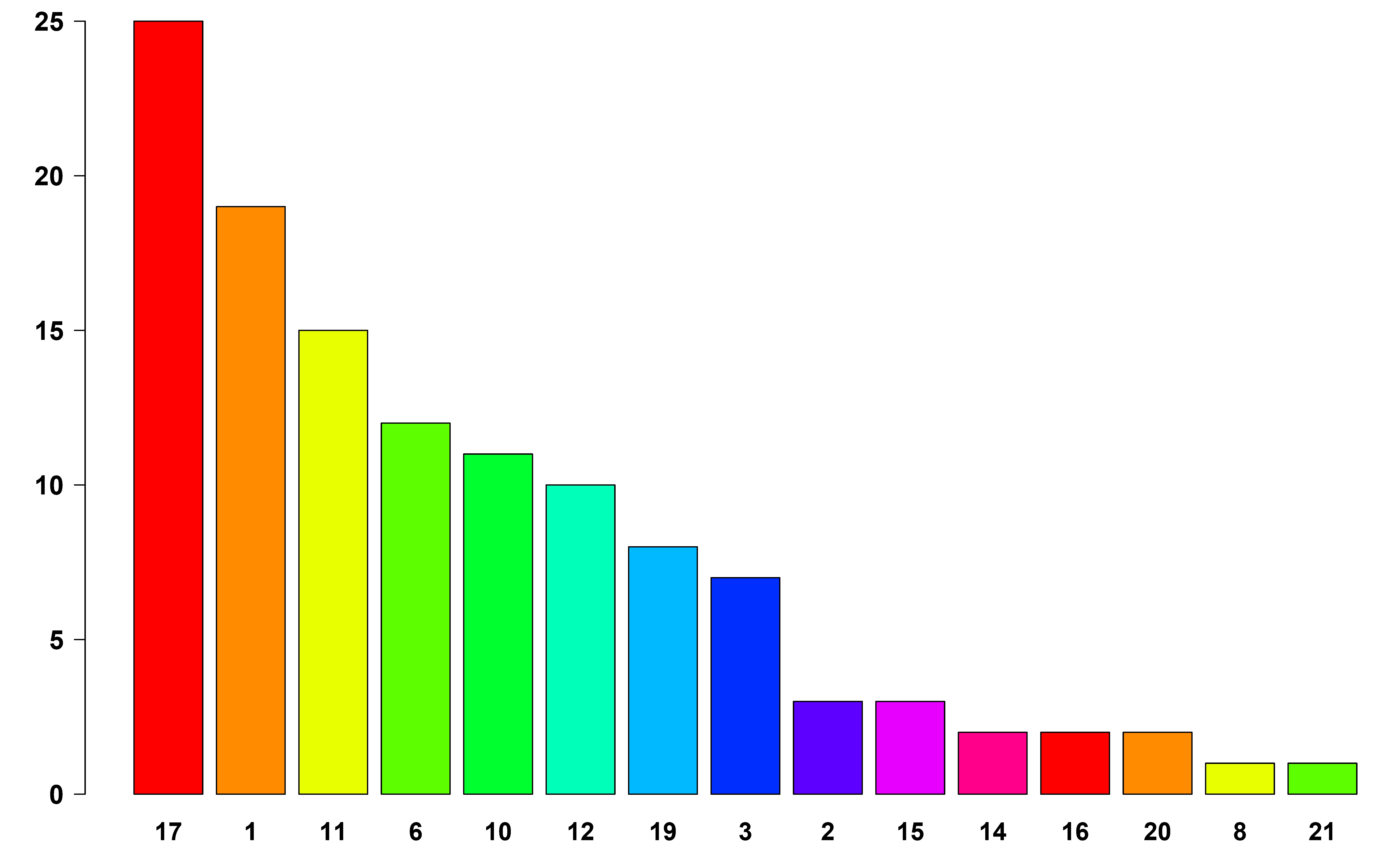


Figure S17. The number of pleiotropic genes in the different chromosome.


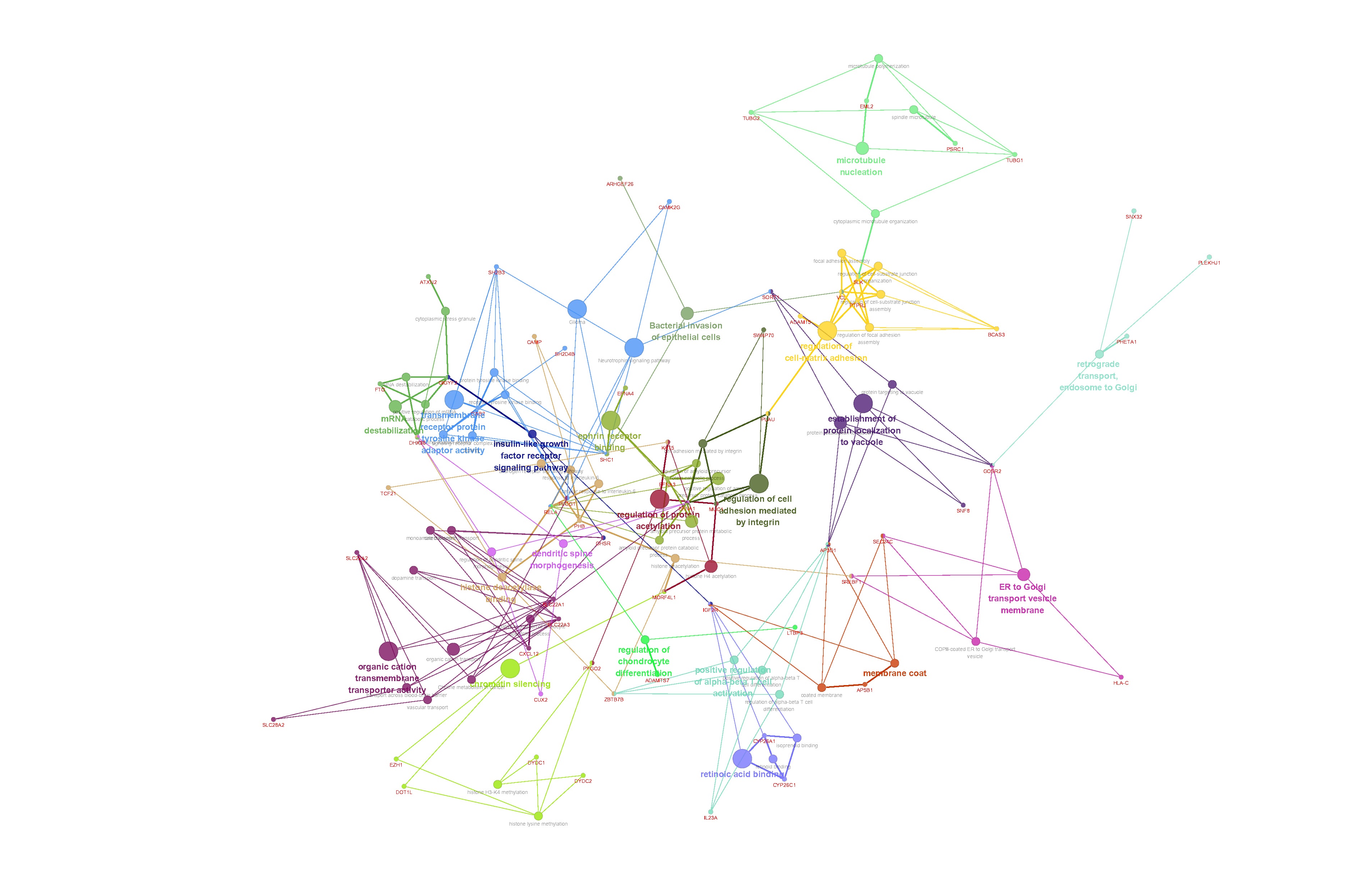


Figure S18. Visualization of KEGG pathways for these newly identified genes with pleiotropic effects. Functionally grouped network with terms as nodes linked based on their kappa score level (≥ 0.3).
